# Symmetric and Asymmetric Dielectric/Metal/Dielectric Electrodes for Semitransparent Perovskite Solar Cells

**DOI:** 10.1021/acsomega.6c07322

**Published:** 2026-09-29

**Authors:** Vittorio Ferrara, Silvia Orecchio, Giuseppe Arrabito, Fabio Principato, Alessandro Auditore, Valentina Spampinato, Antonino Licciardello, Valeria Vetri, Michelangelo Scopelliti, Bruno Pignataro

**Affiliations:** † Department of Physics and Chemistry - Emilio Segrè, 18998 University of Palermo, viale delle Scienze, Palermo 90128, Italy; ‡ Department of Chemical Sciences, University of Catania, viale A. Doria 6, 95125 Catania, Italy

## Abstract

The design of transparent top electrodes is essential for the implementation of advanced photovoltaic technologies, such as building-integrated photovoltaics, agrovoltaics, and tandem devices. In this context, perovskite solar cells implemented with transparent electrodes can emerge as a leading technology due to their tunable optical bandgap and high power conversion efficiency. However, achieving high transparency remains a critical bottleneck, as top electrodes are typically based on opaque noble metal films. This creates an inherent trade-off between electrical conductivity and optical transparency, which limits the overall device performance. In this work, Au-based dielectric/metal/dielectric (D/M/D) multilayers were systematically investigated as top electrodes in both chemically symmetric and asymmetric configurations, employing MoO*_x_* and WO*_x_* as dielectric layers. Through a multitechnique characterization approach, it was demonstrated that the careful design of the selected materials and thicknesses of the Au-based D/M/D constituting layers enables precise tuning of their optical properties. In particular, changing from symmetric to asymmetric configurations provides additional degrees of freedom for light management, enabling the independent modulation of transmittance in the visible and near-infrared spectral regions. The symmetric stacks tend to maximize transmittance in both regions simultaneously, and the asymmetric design allows for selective optimization of transparency in the different spectral ranges. The optimized D/M/D electrodes maintain a low sheet resistance of 4.2 Ω/sq, while reaching transmittance maxima higher than 75%, significantly surpassing standard metallic electrodes. The Au-based D/M/D multilayers herein implemented were successfully integrated as a hole-collecting top electrode into n–i–p semitransparent perovskite solar cells, achieving a power conversion efficiency (PCE) of 13%, comparable to reference devices, but providing significantly enhanced and modulable optical transparency. This strategy establishes Au-based D/M/D architectures as versatile multilayers for next-generation photovoltaics with finely tunable spectrally selective properties.

## Graphical abstract

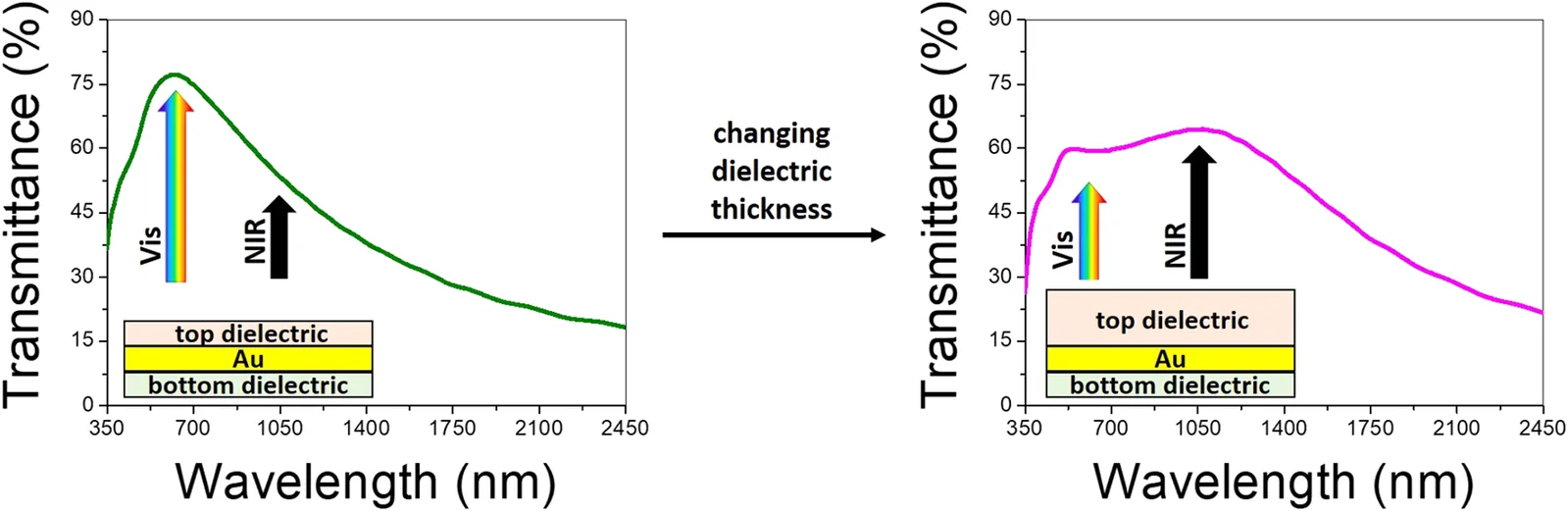

## 1 Introduction

Over the past decade, metal halide perovskite solar cells (PSCs) have emerged as one of the most promising next-generation photovoltaic (PV) technologies, owing to their rapid increase in power conversion efficiency (PCE), low-cost fabrication routes, and high versatility in device engineering.^1–3^ Their outstanding optoelectronic properties, such as high absorption coefficients, long carrier diffusion lengths, and tunable bandgaps,^4–6^ make them ideal candidates for next-generation photovoltaics that can complement the already widely spread use of silicon solar panels. Most of the innovative applications of PSCs depend on the possibility of designing them to be see-through and spectrally selective, thus opening applications that are not feasible with traditional silicon photovoltaics.^7,8^ Indeed, semitransparent PSCs (ST-PSCs) can be engineered to achieve tailored optical transmission across the visible and near-infrared (NIR) spectral regions, enabling their integration in a wide range of applications, including building-integrated photovoltaics (BIPV), agrivoltaics,^9–12^ and tandem photovoltaic architectures with silicon solar cells.^13,14^ However, achieving semitransparency in a solar cell is a complex task, as light absorption is in fact the fundamental process to make the device work.

The optical properties of the active perovskite phase have been intensively investigated to achieve tailored spectral modulation. Perovskites are particularly suitable for this purpose, since it is well established that their spectral characteristics can be tuned by varying the chemical composition resulting in precise bandgap modulation.^15,16^ For the case of lead halide perovskites, emerging in the PV applications, the substitution of the halide ions allows a systematic change in the absorption spectrum of the perovskite film across the visible range from 1.54 to 2.44 eV.^17^ Although A-site cation substitution typically exerts a less direct influence on the electronic band, it can indirectly modulate the bandgap by inducing structural distortions leading to visible or NIR absorption.^18,19^ In turn, the light emission can be modified too, as demonstrated by introducing a controlled excess of Cs^+^ in lead-free double perovskites leading to a stable white-light luminescence.^20^ Alternatively, surface functionalization has been investigated as a powerful strategy to modulate the spectral properties improving also the stability of perovskite materials. Exploring different functional groups for surface binding, it has been established that carboxylates, phosphonates, and sulfonates typically bind to under-coordinated Pb^2+^ cations through chelation or dative covalent bonds, alkylammonium groups interact with surface halide anions via hydrogen-bonding interactions.^21,22^ For example, the application of bidentate ligands such as 2,2′-iminodibenzoic acid has been shown to effectively passivate surface defects in CsPbI_3_ nanocrystals, leading to a near-unity photoluminescence quantum yield of 95% and superior structural stability through a high binding energy of 1.4 eV.^23^ Also, the treatment of perovskite nanostructures with thiophene-2-sulfonamide ligand induces a controlled blue shift in emission by regulating crystal growth.^24^ Therefore, the optical properties of perovskites can work in synergy with their inherent structural and functional versatility to drive innovation across multiple technological fields, ranging from semitransparent photovoltaics to flexible devices^25^ and advanced platforms like tandem catalysis for sustainable energy conversion.^26^

Nonetheless, the transparency of ST-PSCs also depends on the optical features of each layer constituting the whole device. In this respect, the top electrode represents a critical bottleneck, since the trade-off between electrical conductivity and transparency makes the development of transparent electrodes a central challenge for the optimization of ST-PSCs.^27,28^ Among the systems explored to date as electrodes, dielectric/metal/dielectric (D/M/D) multilayers have emerged for high electrical conductivity and visible transparency.^29^ D/M/D exhibits higher transparency than its corresponding single-metal thin films, thanks to the combination of two physical effects. By embedding a thin metal layer with a low refractive index between two high-refractive-index dielectric layers, surface plasmon suppression and optical interference at the metal/dielectric interfaces are promoted, resulting in a significant enhancement of optical transmittance.

The proper choice of the dielectric material, in terms of band structure, work function, and thickness, represents a fundamental aspect that influences both the electrical and optical behavior of the D/M/D stacks. Indeed, the nature of the dielectric layer deposited on the metallic film directly affects the charge distribution at the metal-dielectric interface. Depending on the dielectric material and its intrinsic polarity, charge accumulation can occur at the interface,^30,31^ resulting in interfacial polarization and variations in the local electric field with a magnitude and spatial distribution influenced by the dielectric thickness. These interfacial phenomena can modify the local electrostatic potential and the electronic structure of the multilayer, potentially promoting band bending and altering the transport of charge carriers across the structure.^30^ The dielectric layers can modulate the electric field distribution across the multilayer by acting as field-grading components,^32^ with their dielectric properties and thicknesses determining the extent to which the electric field is redistributed within and across the different layers. Such effects are particularly relevant in thin metallic films, where interfacial charge accumulation and local electric field variations can influence the electrical response and may contribute to electrically induced degradation processes over prolonged operation.

Moreover, selecting dielectric layers with appropriate optical characteristics enables precise regulation of heat exchange by modulating NIR absorption, reflection, and transmission through the modification of the metallic film’s spectral properties.^33^ Furthermore, the use of photocatalytic metal oxides such as TiO_2_ may provide a functional surface leading to self-cleaning properties under sunlight, offering a robust mechanism to combine targeted spectral selectivity with active surface protection.^34–36^ In more advanced systems, heat management and photocatalytic properties have been exploited to achieve synchronized solar energy harvesting and environmental remediation.^37^

A comparison with conventional and emerging transparent conductive electrodes highlights the potential advantages of D/M/D architectures for semitransparent and thermally sensitive optoelectronic devices. Conventional transparent conductive oxides, particularly indium tin oxide (ITO), combine high optical transmittance with low electrical resistivity and currently represent an established choice for transparent electrodes. However, their intrinsic mechanical brittleness and the need for elevated-temperature treatments can limit their integration as top electrodes in thermally sensitive devices, such as ST-PSCs.^38–40^ Alternative transparent conductive systems, including metallic nanowires and carbon nanotubes, provide attractive mechanical flexibility and low-temperature processability, but their implementation can be limited by high surface roughness, junction resistance, or insufficient electrical conductivity for high-performance applications.^41,42^ Conducting polymers represent another flexible alternative, however, they may exhibit material-specific limitations depending on their chemical composition.^43,44^ For example, the well-known polymeric hole transport layer PEDOT:PSS is characterized by intrinsic acidity and hygroscopicity, which can negatively affect the chemical stability of adjacent functional layers and the long-term stability of the device.^45^ Finally, ultrathin continuous metal films, widely used as the top electrode in ST-PSCs, provide excellent electrical conductivity at nanometric thicknesses, but their transparency is quite limited. This aspect can be modulated by sandwiching the ultrathin metallic layer between the dielectric layers, forming a D/M/D multilayer. Indeed, the D/M/D architecture combines the high electrical conductivity and mechanical flexibility of an ultrathin metallic film along with the hole-transport and selective-contact functionality provided by the dielectric oxides.^46^ This is further implemented by the spectral selectivity enabled by the tailored dielectric chemical composition and thickness as well as by the possibility of low-temperature deposition, making these electrodes particularly suitable for integration into thermally sensitive devices, such as ST-PSCs. To the best of our knowledge, record transparency has been reported with the architecture ZnO/Cu-doped Ag/Al_2_O_3_ exhibiting over 90% of transmittance in the visible range.^46^ Many examples of D/M/D systems with application in building fenestration have been reported, especially the Ag-based multilayers have reached commercialization.^47^

The promising and highly tunable properties of D/M/D multilayers have driven their use in the PV context mainly as transparent electrodes.^48^ Several relevant studies have been carried out toward the integration of D/M/D in different kinds of solar cells, obtaining performance comparable with respect to standard electrodes.^49^ Au-based D/M/D structures have been investigated due to the use of Au as a common precious metal in ST-PSCs. A representative example is the MoO_*x*_/Au/MoO_*x*_ multilayer used as a top electrode in a standard ST-PSC with architecture glass/FTO/CH_3_NH_3_PbI_3_/spiro-OMeTAD/MoO_*x*_/Au/MoO_*x*_, reaching a best power conversion efficiency (PCE) of 13% while maintaining transparency.^50^ Additionally, for nanometric thick Au domains, the possibility to independently modulate the spectral features has been demonstrated by combining the properties of metals with semiconductors and dielectrics. For instance, the implementation of precisely designed structures of Au of nanometric thickness has been shown to enable sophisticated and synchronized manipulation of wavefront and spectral responses across different domains, reinforcing that strategic structural arrangement is a fundamental tool for achieving targeted selectivity.^51^ Ag-based D/M/D top electrodes have also been widely explored, showing good device performance and transparency, including in flexible solar cells.^52^

The D/M/D architectures based on metal oxides have already demonstrated their potentiality even in tandem photovoltaic devices. Some interesting strategies widely pursued rely on the use of ITO or on organic self-assembled monolayers to optimize energy-level alignment and interfacial passivation. For instance, composite self-assembled monolayers have recently enabled Perovskite/TOPCon tandem solar cells to reach efficiencies over 32%.^53^ Sputtered ITO has been validated as a high-performance recombination layer for monolithic Perovskite/Si tandem devices.^54^ In this context, D/M/D architectures integrated in tandem devices offer an additional degree of functionality, enabling spectral selectivity while simultaneously providing efficient charge transport. The multilayer MoO_*x*_/Au seeds/Ag/MoO_*x*_ structures have been used as outer electrodes in CIGS–perovskite 4-terminal tandem solar cells.^55^ In addition, D/M/D layers have been implemented as intermediate contacts between subcells in tandem devices, enhancing transparency and reducing optical losses between the top and bottom cells.^56^

Notably, most of the reported D/M/D electrodes employed in semitransparent solar cells adopt chemically symmetric configurations, whereas asymmetric D/M/D architectures remain largely unexplored. An example of chemically asymmetric D/M/D structure is TiO*_x_*/Ag/AZO multilayer which has demonstrated excellent electrical conductivity and optical transparency.^57^ Another example is ZnS/Ag/WO_3_ which has been exploited as asymmetric electrode in organic light-emitting diode.^58^ The ZnS/Ag/TiO_2_ multilayer electrode has also demonstrated very high visible-light transparency with a state-of-the-art maximum transmittance of 90.23% at 460 nm and a sheet resistance of 5 Ω/sq.^59^ Nevertheless, these systems have not yet been integrated into solar cells. Few examples of asymmetric D/M/D integration in ST-PSCs have been reported mainly based on the use of Ag as metallic layer. For instance, the system MoO_*x*_/Ag/WO_*x*_ has been integrated in ST-PSCs, with different lead halide perovskites as photoactive layers, achieving promising results.^60,61^ Another interesting example of asymmetric D/M/D based on Ag has been reported in a ST-PSC completed with MoO_*x*_/Ag/ZnS with a particular attention to the NIR reflecting properties and a PCE of 13%.^62^

In addition, multilayer stacks incorporating dielectric materials have also been investigated as protective structures for optoelectronic devices, exploiting the intrinsic barrier properties of both organic^63^ and inorganic dielectric layers^64^ against environmental degradation. Among these architectures, D/M/D multilayers are particularly attractive because the dielectric layers can provide both optical modulation and protective functionalities, while the metal layer ensures high electrical conductivity. For example, a b-MoO_*x*_/Ag/t-MoO_*x*_ multilayer was demonstrated to simultaneously act as a top electrode and an encapsulation layer, providing an external protective barrier against the ingress of moisture and oxygen from the surrounding environment. Owing to this multifunctional D/M/D top electrode, the resulting n–i–p PSCs retained 92% of their initial current density after 24 h of continuous operation under ambient conditions.^52^

Overall, chemically symmetric D/M/D multilayers have been widely investigated due to their combination of high electrical conductivity and excellent transparency in the visible range, while the asymmetric architectures have received considerably less attention. The potential of asymmetric structures to provide independent control over different regions of the solar spectrum has not yet been systematically explored, and a direct comparison with chemically symmetric D/M/D architectures is still lacking. In particular, the influence of introducing chemical and/or geometrical asymmetry in the dielectric layers on the spectral distribution of transmittance remains insufficiently understood. Addressing this gap is essential for determining whether asymmetric architectures can offer additional degrees of freedom for tailoring the optical response beyond those achievable with conventional symmetric stacks. Therefore, a systematic comparison between symmetric and asymmetric Au-based D/M/D configurations is undertaken in this work to elucidate the distinct optical functionalities of the two architectures and to assess the potential of asymmetry as a strategy for independently tuning the visible and near-infrared regions of the solar spectrum. More specifically, chemically symmetric and asymmetric D/M/D multilayers were developed with structure b-D/Au/t-D, where b-D indicates the bottom dielectric, and t-D the top dielectric. The herein reported b-D/Au/t-D multilayer electrodes were systematically investigated to elucidate the role of dielectric chemical composition and thickness in governing electrical and optical properties of the multistacks. In particular, Au was selected for its superior chemical stability with respect to Al and Ag.^65^ The metal oxides MoO_*x*_ and WO_*x*_ were selected as dielectrics due to their high work functions (4.7–6.4 eV), which promote efficient hole extraction from spiro-OMeTAD, the standard organic hole-transport layer in n-i-p PSCs.^66,67^ In terms of thermal stability, thin films of substoichiometric MoO_*x*_ and WO_*x*_ are expected to preserve their charge transport functionality within the temperature range relevant to PV applications, typically up to ∼85 °C under severe operating conditions. Previous studies showed that the oxidation state of tungsten in WO_*x*_ remains stable above 327 °C,^68^ indicating its remarkable thermal stability, whereas MoO_*x*_ exhibits comparatively higher thermal sensitivity,^69^ which can be exploited to enhance its electrical conductivity, as increasing temperature promotes oxygen-vacancy formation.^70^ These vacancies introduce donor states below the Fermi level, increasing the concentration of electrically active charge carriers and thereby enhancing charge transport. The effect of the dielectric materials and their thickness on the morphology and optical features of the ultrathin Au layer was investigated, providing insights into the structure and resulting properties of the D/M/D multilayers. The fabrication and multitechnique characterization of chemically symmetric and asymmetric D/M/D architectures were deeply explored to tune the visible and NIR optical response while preserving suitable electrical performance. Finally, the optimized b-D/Au/t-D structures were integrated as TTEs in ST-PSCs, demonstrating their suitability as transparent electrodes for semitransparent photovoltaic devices.

## 2 Materials and Methods

### 2.1 Materials

Methylammonium iodide (CH_3_NH_3_I, 99.9%) was purchased from Ossila, lead chloride (PbCl_2_, 99.999%), and gold (Au, 99.999%) were obtained from Alfa Aesar, titanium­(IV) isopropoxide (99.999%), molybdenum trioxide (MoO_3_, 99.97%), tungsten trioxide (WO_3_, 99.995%), 4-tertbutylpyridine (TBP, 96%), lithium bis­(trifluoromethylsulfonyl)­imide (LiTFSI, 99.95%), tris­(2-(1*H*-pyrazol-1-yl)-4-*tert*-butylpyridine)­cobalt­(III) tri­[bis­(trifluoromethane)­sulfonimide] (FK 209, 98%), anhydrous chlorobenzene (CB, 99.8%), anhydrous acetonitrile (ACN, 99.8%), *N*,*N*-dimethylformamide (DMF, anhydrous, 99.8%), and anhydrous isopropanol (IPA, 99.5%), 2,20,7,70-tetrakis­(N,N-diphenylaminophenyl)-9,9-spirobi-fluorene (Spiro-OMeTAD) were supplied by Sigma-Aldrich. All chemicals were used as received without further purification. Fluorine-doped tin oxide (FTO)-coated glass slides (TEC7) were purchased from Visiontek Systems.

### 2.2 b-D/Au/t-D Deposition and Characterization

The Au-based D/M/D multilayers were fabricated with the structure b-D/Au/t-D, where b and t indicate the bottom and top dielectric layer, respectively. The b-D/Au/t-D multilayers were deposited by thermal evaporation with the b-D bottom dielectric layer 6 nm thick, the Au layer of 10 nm and the t-D top dielectric layer of thickness ranging from 0 to 40 nm for MoO_*x*_, and from 0 to 70 nm for WO_*x*_, on soda lime glass. As dielectric layers, MoO_3_ and WO_3_ powders were used as starting materials, and in both cases the evaporation rate was set to 0.3 Å/s. For the deposition of Au, the evaporation rate was 0.9 Å/s. All the layer thicknesses were monitored during the thermal deposition through a calibrated quartz crystal microbalance placed inside the evaporation chamber. All the possible combinations of b-D/Au/t-D were prepared, that is, the chemically symmetric b-MoO_*x*_/Au/t-MoO_*x*_ and b-WO_*x*_/Au/t-WO_*x*_, and chemically asymmetric b-MoO_*x*_/Au/t-WO_*x*_ and b-WO_*x*_/Au/t-MoO_*x*_ multilayers.

XPS spectra were acquired by means of a PHI 5000 VersaProbe II spectrometer (ULVAC-PHI) using an Al anode (Al Kα_1_ = 1486.6 eV), pass energy 23.500 eV, energy step 0.05 eV. All spectra were collected under charge compensation of both electrons and Ar+ ions. The illuminated area was 100 μm in diameter; the analyzer operated in FAT mode. Energy values are reported using the adventitious carbon peak (284.80 eV) as a reference. Data were analyzed with MultiPak software, version 9.9.3 (ULVAC-PHI), using a Shirley background and a pseudo-Voigt line shape for peak fitting. The AFM images were acquired in air by means of a Bruker Dimension FastScan (Bruker Corporation, Billerica, MA). The scans were recorded in tapping mode by using FAST-SCAN-A probes with apical radius of about 5 nm and 512 × 512 points per image. The images of 2 μm × 2 μm area were analyzed by Gwyddion 2.70.^71^

Time-of-flight secondary ion mass spectrometry (ToF-SIMS) depth profiling was conducted using a ToF-SIMS IV instrument (IONTOF GmbH, Münster, Germany) operated in dual-beam mode. Sputtering was performed with a Cs^+^ ion source at an acceleration energy of 1 keV and a beam current of 12 nA. The analysis was carried out using a Bi_3_^+^ primary ion beam operated at 25 keV with a pulsed current of 0.07 pA, optimized to achieve high mass resolution while minimizing sample damage. The analysis beam was rastered over a 30 μm × 30 μm area, aligned concentrically within the sputter crater of 200 μm × 200 μm. Depth profiling was performed in the so-called “non-interlaced” mode, where sputter and analysis cycles were alternated to generate a depth profile, with secondary ion signals collected after each sputter step. The b-D/Au/t-D stack homogeneity and thickness were evaluated by scanning electron microscopy (SEM) using a Phenom ProXSEM. The as-deposited samples were analyzed in top-view and cross section at different magnifications. Energy-dispersive X-ray spectroscopy (EDS) analysis was performed using a scanning electron microscope, ESEM Philips XL30 (Massachusetts, USA). The b-D/Au/t-D optical characterization was carried out by recording the UV–vis-NIR transmittance and reflectance spectra over the spectral range 350–2500 nm using a V-770 Jasco spectrophotometer. A 60 mm integrating sphere (ISN-923) was positioned inside UV–vis-NIR spectrophotometer for reflectance measurements. Sheet resistance of the D/M/D structures was measured by means of a Keithley 2400 source-meter unit equipped with four probes for electrical contact with the surface.

### 2.3 Solar Cells Fabrication and Characterization

FTO-coated glass slides (TEC7) were cleaned by sonication in acetone and IPA for 15 min each, then dried with anhydrous N_2_, and exposed to ultraviolet-ozone treatment for 40 min. After that, a compact titania (c-TiO_2_) electron transport layer was synthesized on the FTO surface by a sol–gel process starting from a mildly acidic solution of 2 M titanium­(IV) isopropoxide in IPA (175 μL in 2.5 mL) acidified with 17.5 μL of 2 M aqueous HCl, and spin-coated through a 0.2 μm PTFE filter onto the FTO substrate at 2000 rpm for 60 s. The obtained film was dried for 10 min at 125 °C on a hot plate in air and calcined at 500 °C for 45 min, then cooled down to room temperature overnight. The substrates coated with c-TiO_2_ were then transferred into a N_2_-filled glovebox system (MBraun, Germany; O_2_ < 0.1 ppm and H_2_O < 0.1 ppm) for all the next fabrication steps. On the c-TiO_2_ layer, a film of CH_3_NH_3_PbI_3–x_Cl_*x*_ (MAPIC) perovskite was synthesized by spin coating 140 μL of a solution of CH_3_NH_3_I and PbCl_2_ in a 3:1 molar ratio in anhydrous DMF, typically 398 mg of CH_3_NH_3_I and 237 mg of PbCl_2_ in 1 mL of DMF in anhydrous DMF at 4000 rpm for 45 s. The film obtained was annealed on a hot plate at 105 °C for 1.5 h. Then, the hole transport layer was deposited from a solution of 60 mM p-doped spiro-OMeTAD dissolved in CB, typically 72.3 mg of spiro-OMeTAD added with 28.8 μL of TBP, 17.5 μL of a LiTFSI (520 mg/mL), and 10 μL of FK 209 in 1 mL of CB. The solution was spin-coated on the perovskite layer at 4000 rpm for 45 s. The devices were then stored overnight inside a desiccator in air. Finally, the D/M/D top electrodes were thermally evaporated, as described in the previous section, onto the FTO/c-TiO_2_/CH_3_NH_3_PbI_3–x_Cl_*x*_/spiro-OMeTAD surfaces through a metallic mask under a vacuum of 5 × 10^–6^ mbar to get circular cells with diameters of 2, 3, and 4 mm. Reference solar cells were fabricated by depositing a standard D/M layer b-MoO_*x*_/Au. The I–V characteristics of the solar cells were acquired using a Keithley 2400 source-measure unit. The devices were illuminated from the FTO side by a Sunlite Solar Simulator (model 11002, Class ABA, Abet Technologies) equipped with a 100 W xenon arc lamp, an AM 1.5G filter, and an irradiance power of 100 mW/cm^2^ corresponding to 1 Sun. For calibration, a commercial Si reference cell was used (Abet Technologies, model 15151). All J-V measurements were performed in a glovebox at 25 °C using a Keithley 4200 source-measure unit.

## 3 Results and Discussion

The D/M/D developed in this work were fabricated following a b-D/Au/t-D configuration, where the bottom (b-D) and top (t-D) dielectric layers consist of either MoO_*x*_ or WO_*x*_. These electrodes were subsequently integrated into ST-PSCs with an n–i–p architecture, as illustrated in Figure 1A. In particular, the structure of ST-PSCs consists of an n–i–p device fabricated on glass coated with fluorine-doped tin oxide (FTO) as the transparent bottom electrode, compact titanium dioxide (c-TiO_2_) as the electron transport layer, the hybrid organic/inorganic perovskite methylammonium lead mixed iodide/chloride CH_3_NH_3_PbI_3_–_ *x*_Cl_*x*_ (MAPIC) as the light absorber, well-known for its optoelectronic properties and remarkable photovoltaic performance, and p-doped spiro-OMeTAD as the hole transport layer. The device was completed with the b-D/Au/t-D as a transparent top electrode to test their compatibility and performance in ST-PSCs. The b-D/Au/t-D top electrodes herein developed and tested are schematically reported in Figure 1B. The multilayers rely on the use of a Au thin film as the conductive core, selected as a well-performing and widely used metal in PSCs. The Au thin film was sandwiched between layers of MoO_*x*_ or WO_*x*_, exploring all the possible chemical configurations. Both chemically symmetric and asymmetric multilayer stacks were fabricated by varying the nature of the dielectric layers. Specifically, the symmetric and asymmetric configurations are b-MoO*_x_*/Au/t-MoO*_x_* or b-WO*_x_*/Au/t-WO*_x_* and b-WO*_x_*/Au/t-MoO*_x_* or b-MoO*_x_*/Au/t-WO*_x_*, respectively. In this study, the optimization of the b-D/Au/t-D multilayer architecture followed specific criteria to ensure both a systematic investigation of the spectral properties of the multilayers as well as a high electrical performance for further integration in ST-PSCs. The thickness of the b-D layer was fixed at 6 nm for all configurations, since a 6 nm thick b-MoO_*x*_ layer has demonstrated suitable features as both a growth layer for Au and as an effective anode-buffer layer for hole extraction in n-i-p ST-PSCs.^72,73^ For a direct comparison between the dielectrics, the b-WO_*x*_ thickness was set to the same value of 6 nm. The Au layer thickness was fixed at 10 nm as this value represents the optimal threshold to balance electrical conductivity and optical transparency. Au films thinner than 10 nm undergo a sharp drop in their electrical performance, making them unsuitable as top electrodes for ST-PSCs. Finally, the t-D thickness was systematically varied to maximize transparency in the visible and NIR range. Following typical approaches reported in the literature,^49^ the t-D thickness was increased in steps of 10 nm, ranging from 0 to 40 nm for t-MoO_*x*_ and from 0 to 70 nm for t-WO_*x*_. This systematic variation allowed for a precise evaluation of the spectral changes during film growth, enabling the selection of the most appropriate thickness to optimize visible and NIR transmittance.

**Figure 1:**
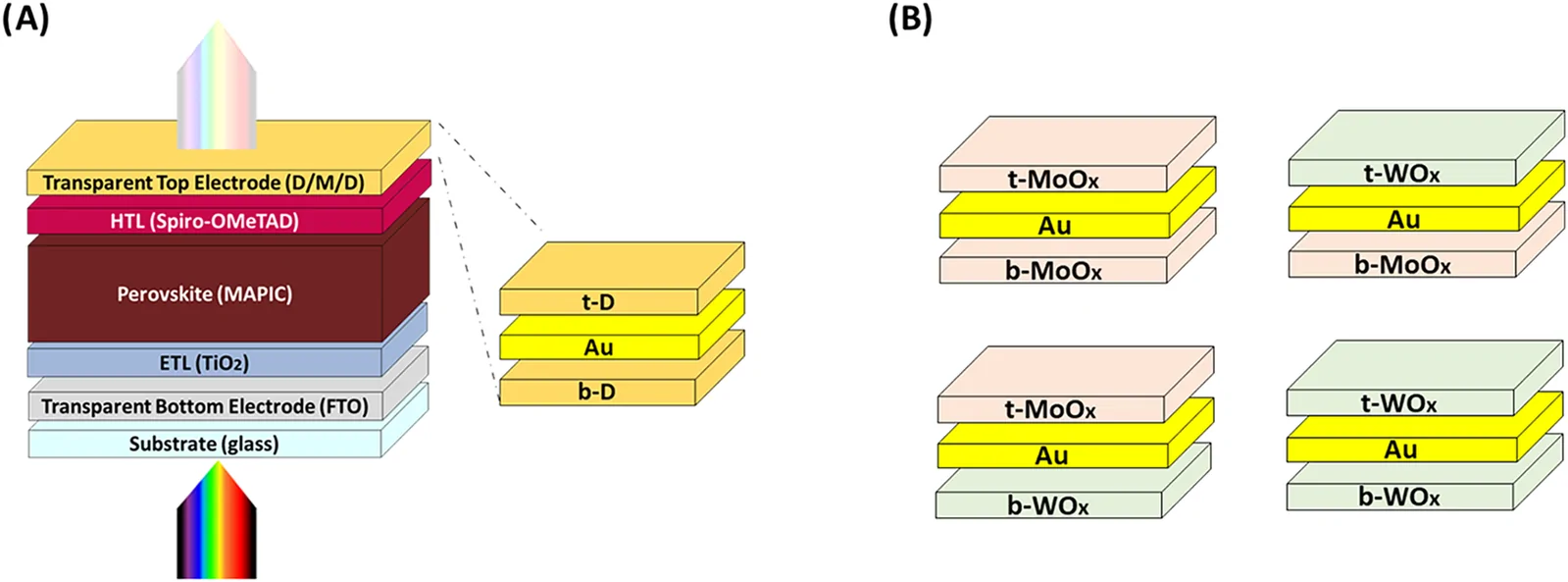
Schematic representation of semitransparent perovskite solar cells (ST-PSCs) integrated with dielectric/metal/dielectric (D/M/D) transparent top electrodes: (A) n–i–p device architecture, constituted by glass as substrate, fluorine-doped tin oxide (FTO) as the transparent bottom electrode, compact titanium dioxide (c-TiO_2_) as the electron transport layer (ETL), CH_3_NH_3_PbI_3–*x*_Cl_*x*_ (MAPIC) perovskite as the light absorber, p-doped spiro-OMeTAD as the hole transport layer (HTL), completed with a b-D/Au/t-D top electrode where bottom dielectric (b-D) and top dielectric (t-D) layers represent either MoO_*x*_ or WO_*x*_. (B) Detailed schematics of the four explored configurations: chemically symmetric multilayers b-MoO*_x_*/Au/t-MoO*_x_* and b-WO*_x_*/Au/t-WO*_x_*, and asymmetric multilayers b-MoO*_x_*/Au/t-WO*_x_* and b-WO*_x_*/Au/t-MoO*_x_*.

### 3.1 Chemical and Structural Characterizations of b-D/Au/t-D Multilayers

The chemical and structural properties of the b-D/Au/t-D multilayers investigated in this work were systematically analyzed to assess both the chemical composition and the degree of interfacial planarity of the stacks deposited via thermal evaporation. To this end, complementary characterization techniques were employed to correlate compositional properties with interface quality, enabling a comprehensive evaluation of the relationship between deposition conditions, material properties, and the resulting structural integrity of the multilayer system. First, the b-D/Au/t-D surface properties were studied. A detailed investigation of the stoichiometry and oxidation states of the deposited films of MoO_*x*_ and WO_*x*_ was carried out by X-ray photoelectron spectroscopy (XPS). XPS has been employed with the aim of revealing the stoichiometry modification of the thin film by the thermal evaporation and recombination processes at the substrate interface. Figure 2 reports the XPS high-resolution spectra of Mo and W. The Mo 3d and W 4f and 5p signals display well-defined peak systems that were fitted with the components summarized in Table 1. For the MoO*_x_* thin film, the Mo 3d spectrum consists of two main doublets corresponding to different oxidation states. The lower binding energy component, with the peaks Mo 3d_5_/_2_ at 231.92 eV and Mo 3d_3_/_2_ at 235.05 eV, is assigned to Mo­(V) and represents a minor contribution (8.05%). The higher binding energy doublet, located at 233.06 and 236.19 eV, is attributed to Mo­(VI) and accounts for the dominant fraction (91.95%). Similarly, the WO*_x_* thin film shows characteristic W 4f and W 5p signals. The W­(V) component is identified by the peaks W 4f_7_/_2_ at 34.80 eV, W 4f_5_/_2_ at 36.98 eV, and W 5p_3_/_2_ at 40.50 eV, contributing 10.64% to the total spectral weight. The predominant contribution arises from W­(VI), with peaks at 36.18, 38.36, and 42.14 eV, corresponding to 89.36% relative abundance. Overall, XPS analysis highlights that the dielectric thin films are predominantly composed of MoO_3_ (Mo­(VI)) or WO_3_ (W­(VI)), with minor substoichiometric contributions of Mo­(V) and W­(V).

**Figure 2:**
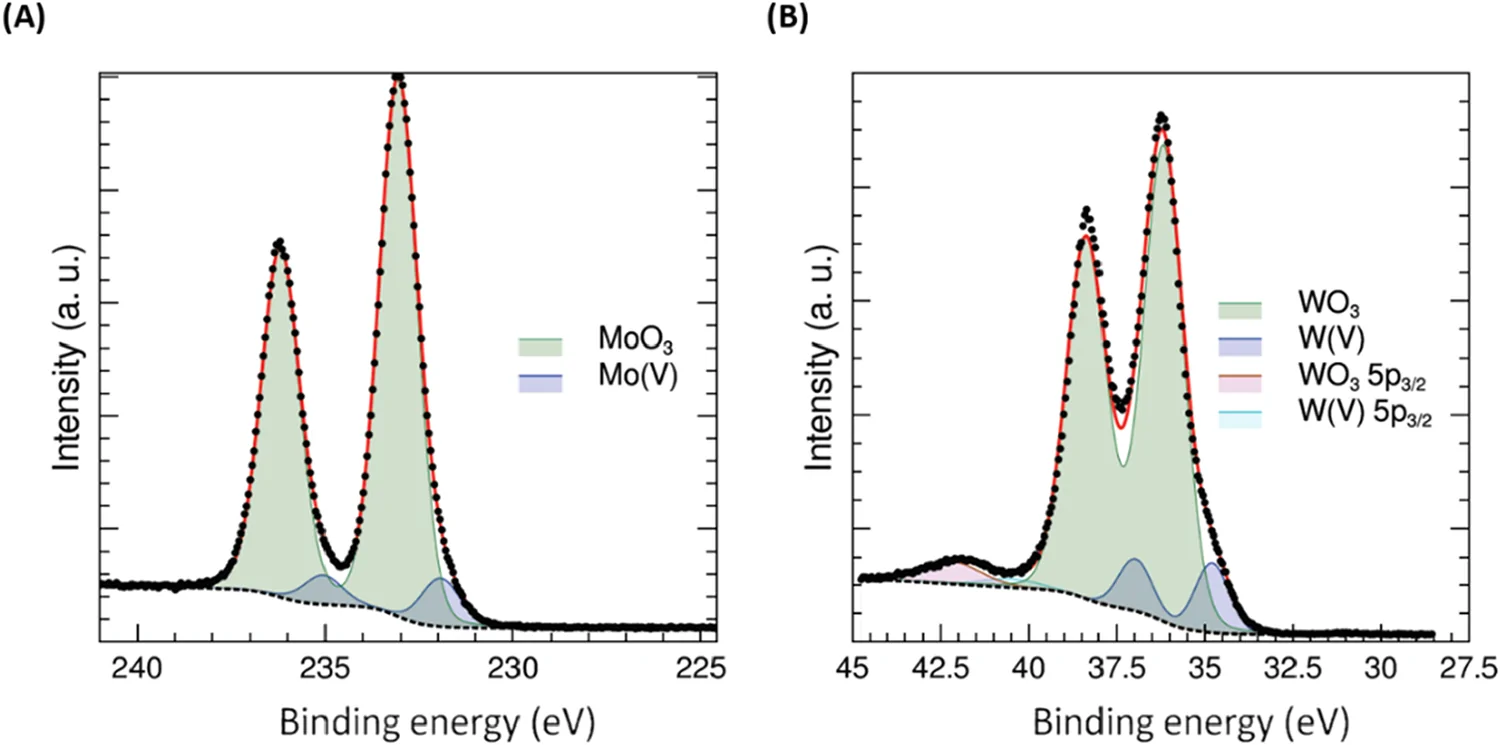
High-resolution X-ray photoelectron spectroscopy (XPS) spectra of Mo 3d for MoO*_x_*, and W 4f and W 5p for WO*_x_* thin films. (A) The Mo 3d spectrum is fitted with two doublets assigned Mo 3d_5/2_ and Mo 3d_3/2_ at 231.92 and 235.05 eV, respectively, for Mo­(V) (relative abundance 8.05%) and at 233.06 and 236.19 eV for Mo­(VI) (relative abundance 91.95%). (B) W spectra showing contributions from W­(V), with peaks at 34.80 eV (4f_7/2_), 36.98 eV (4f_5/2_), and 40.50 eV (5p_3/2_) (relative abundance 10.64%), and W­(VI) with peaks at 36.18, 38.36, and 42.14 eV (relative abundance 89.36%).

**Table 1:** Binding Energy (B.E.) Values and Relative Abundances of the Mo 3d, W 4f, and W 5p Components Obtained from High-Resolution XPS Spectra of MoO*_x_* and WO*_x_* Films 10 nm Thick

MoO*_x_*	Mo 3d_5/2_	Mo 3d_3/2_	relative abundance (%)
Mo(V)	231.92	235.05	8.05
MoO_3_	233.06	236.19	91.95

WO*_x_*	W 4f_7/2_	W 4f_5/2_	W 5p_3/2_	relative abundance (%)
W(V)	34.80	36.98	40.50	10.64
WO_3_	36.18	38.36	42.14	89.36

A preliminary assessment of the surface morphology of Au deposited on b-MoO_*x*_ and b-WO_*x*_ was performed by scanning electron microscopy (SEM) analysis (Figure S1). The top-view SEM images revealed a relatively homogeneous and compact film in the former case, whereas a more heterogeneous and porous morphology was observed for Au deposited on b-WO_*x*_ at the microscale.

The morphological features of each thin film constituting the b-D/Au/t-D stacks were deeply analyzed by atomic force microscopy (AFM). The representative AFM measurements on 2 μm × 2 μm regions are reported in 3D in Figure 3, which highlights the changes in the surface morphology occurring during the stack fabrication. Images were acquired at each step of the deposition during the progressive deposition of b-D/Au/t-D. The first layers of b-MoO_*x*_ (A) and b-WO_*x*_ (B) exhibit both cluster-like surfaces, with an average grain size smaller than 20 ± 5 nm. On these surfaces, a 10 nm thick Au layer was deposited, leading to stacks with a relevant difference in morphology. A more homogeneous, uniform, and continuous film is observed in the case of b-MoO_*x*_/Au, whereas b-WO_*x*_/Au appears to be characterized by the presence of pores of 100 ± 20 nm and a mean depth of 15 ± 5 nm. After investigating the surface morphology of b-D and b-D/Au layers, the morphology of the t-D layer in the four b-D/Au/t-D multilayers was also analyzed. The AFM measurements were performed on samples with the top dielectric layer at a thickness of 40 nm. The images reveal that all the multilayer structures exhibit homogeneous and continuous surface morphologies upon the deposition of the t-D. The sample morphology is homogeneous and flat, with an average grain size of 15 ± 5 nm and a root-mean-square (RMS) roughness of 2 nm or less (Table TS1), with no holes or discontinuities observed. This indicates that the deposition of the top dielectric layer leads to the formation of a continuous coating that effectively covers the underlying layers, resulting in comparable surface morphologies across all the investigated multilayer systems. Also, in the case of the b-WO_*x*_/Au systems show randomly distributed holes, whereas the deposition of the top oxide layer leads to the formation of a homogeneous and continuous surface.

**Figure 3:**
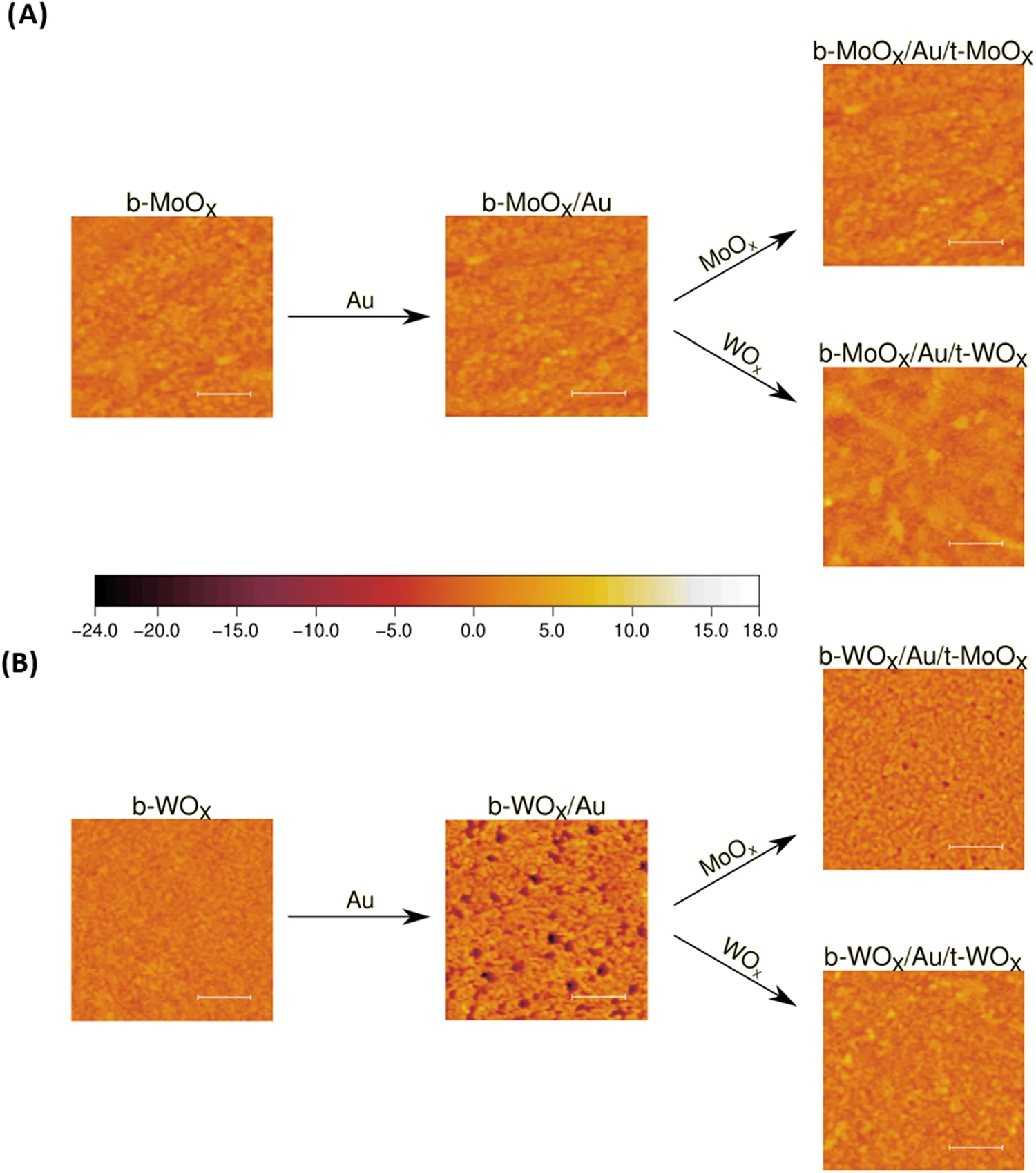
2 μm × 2 μm atomic force microscopy (AFM) images illustrating surface morphology changes during the sequential assembly of b-D/Au/t-D multilayers. (A) Surface morphology of the stack starting from a b-MoO_*x*_ bottom dielectric up to the complete b-MoO_*x*_/Au/t-MoO_*x*_ and b-MoO_*x*_/Au/t-WO_*x*_; (B) Surface morphology of the stack starting from a b-WO_*x*_ bottom dielectric up to the complete b-WO_*x*_/Au/t-MoO_*x*_ and b-WO_*x*_/Au/t-WO_*x*_. The images show the cluster-like dielectric surface of the b-D layers 6 nm thick, the 10 nm thick Au layer on b-MoO_*x*_ and b-WO_*x*_, and the final 40 nm t-D layer. Scale bars are 500 nm. The horizontal color map represents the surface height in nm with respect to the average plane.

Structural properties of the b-D/Au/t-D multilayers through their bulk architecture were then examined. First, a cross-sectional SEM analysis was carried out coupled with energy-dispersive X-ray spectroscopy (EDS) measurements (Figures S2–S6). This investigation confirmed the presence of the deposited b-D/Au/t-D layer with a thickness below 100 nm for all the reported samples. According to the previous characterizations, the D/M/D structures appeared well-defined, dense, and uniform in the case of systems incorporating b-MoO_*x*_ (Figure S2A,B), whereas the b-WO_*x*_-based systems resulted in a discontinuous growth of the Au layer (Figure S2 C–D) as revealed by the less homogeneous morphology and the reduced contrast at the interfaces. The elemental composition obtained by EDS confirmed the presence of Au at the surface of the samples, since Au is readily detectable by electron-beam analytical techniques and often used as a standard. (Figures S3–S6) The Au signal was detected at a higher percentage in the D/M/D architectures where the Au layer was grown on b-MoO_*x*_ compared to those based on b-WO_*x*_, in agreement with the more homogeneous and compact morphology observed in the former case and the more porous and inhomogeneous morphology found in the latter. Concerning the other elements detected, no distinct signals from Mo or W were observed, as the EDS signal is dominated by the chemical composition of the glass substrate due to the relatively large interaction volume of this bulk-sensitive technique compared with the nanometric thickness of the D/M/D layers. A more detailed and surface-sensitive analysis of the Au-based multilayers was carried out through the stack by the Time-of-Flight Secondary Ion Mass Spectrometry (ToF-SIMS) depth profiles with the aim of assessing the uniformity and planarity of the metal/dielectric interfaces. Figure 4 reports the ToF-SIMS depth profiles of the samples with 80 and 20 nm thick t-MoO_*x*_ and t-WO_*x*_ layers, respectively. The signals of the MoO_2_^–^, Au_3_^–^, and WO_3_^–^ ions were monitored to obtain the chemical profiles of the D/M/D multilayers. The Si^–^ signal was used as a marker of the glass substrate interface.

**Figure 4:**
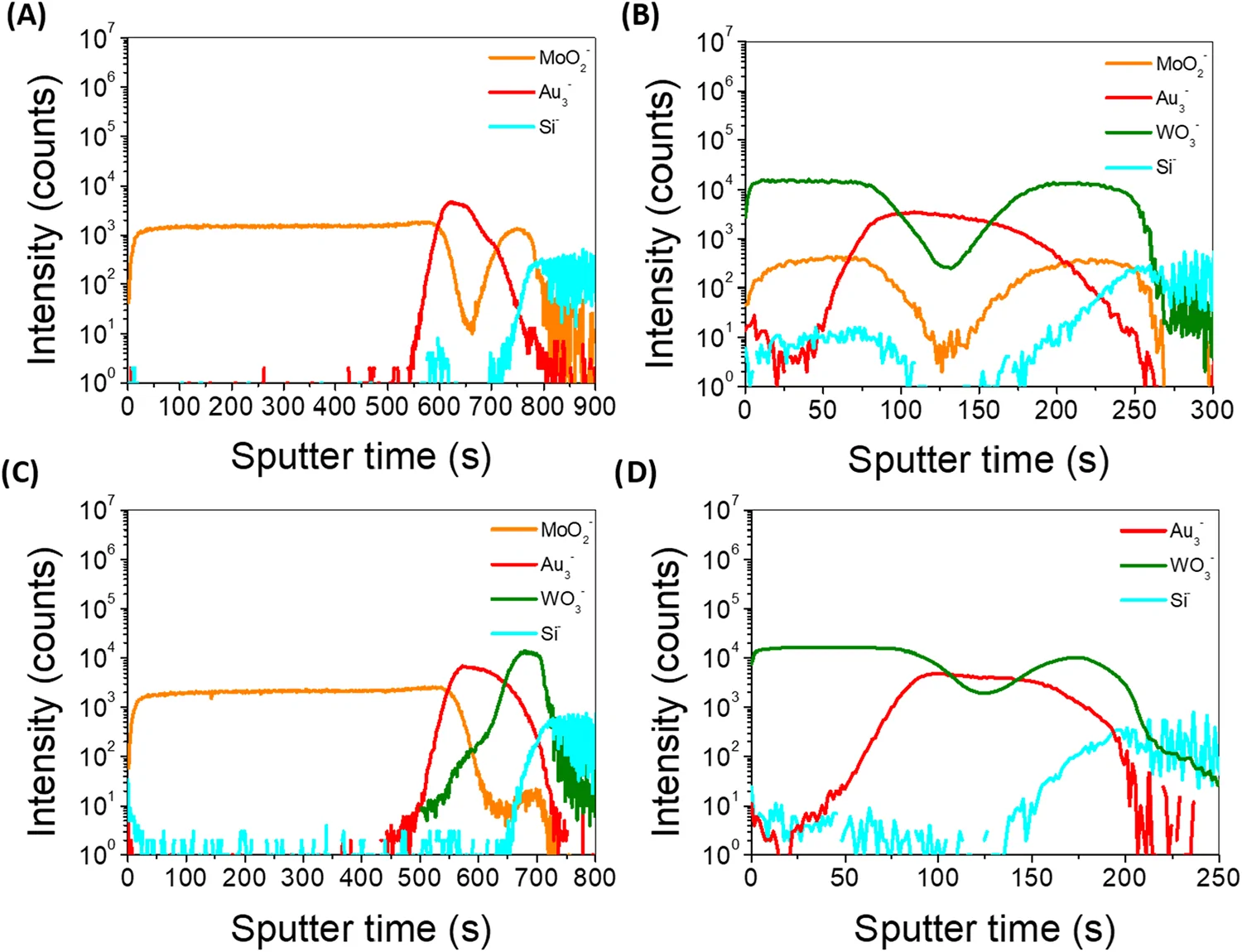
Time-of-flight secondary ion mass spectrometry (ToF-SIMS) depth profiles showing the chemical stratification of (A) b-MoO_*x*_/Au/t-MoO_*x*_, (B) b-MoO_*x*_/Au/t-WO_*x*_, (C) b-WO_*x*_/Au/t-MoO_*x*_, and (D) b-WO_*x*_/Au/t-WO_*x*_ multilayers as a function of sputtering time. Ion signals monitored include MoO_2_^–^ (orange), Au_3_^–^ (red), and WO_3_^–^ (green), with Si^–^ (light blue) marking the glass substrate interface. For all multilayers, the b-D layers are 6 nm thick, the Au layer is 10 nm thick, and t-MoO_*x*_ and t-WO_*x*_ layers are 40 and 20 nm thick, respectively.

The ToF-SIMS depth profiles qualitatively highlight the chemical stratification of the different layers constituting the investigated b-D/Au/t-D structures. For the b-MoO_*x*_/Au/t-MoO_*x*_ and b-WO_*x*_/Au/t-MoO_*x*_ systems of Figure 4A,4C with 80 nm thick t-MoO_*x*_, the layers exhibit well-defined planar interfaces and a suitable stratification consistent with the designed multilayers. It is noteworthy that, in the case of b-WO_*x*_/Au/t-MoO_*x*_, a slight overlap between the Au and WO_*x*_ signals is observed compared with the b-MoO_*x*_-based multilayers. This observation is consistent with AFM results, revealing the presence of superficial holes when Au is deposited on b-WOx, which are absent in the case of b-MoO_*x*_-based multilayers. On the other hand, the b-MoO_*x*_/Au/t-WO_*x*_ and b-WO_*x*_/Au/t-WO_*x*_ multilayers have been investigated with 20 nm thick t-WO_*x*_, as shown in Figure 4B,4D. The b-WO_*x*_/Au/t-WO_*x*_ multilayer shows an almost stratified sequence, whereas an evident overlap of the bottom and top oxide signals through the Au layer is observed for b-MoO_*x*_/Au/t-WO_*x*_. This last observation suggests a partial interfacial mixing more likely induced by the sample overheating during the thermal evaporation of relatively thick t-WO_*x*_. In the b-WO_*x*_/Au/t-WO_*x*_ multilayer, this effect cannot be directly resolved due to the identical chemical nature of the bottom and top layers; however, the depth profile, showing Au intermixed with tungsten oxide, is consistent with significant interfacial intermixing also in this case. Also, the Au signal exhibits a comparable thickness (about 200 s of sputtering time) across all investigated samples, indicating that Au is almost stable against uncontrolled diffusion within the oxide layers.

### 3.2 Optical and Electrical Features of b-D/Au/t-D Multilayers

A key point toward the application of b-D/Au/t-D multilayers as a transparent top electrode in ST-PSCs is the evaluation of their optical properties with a special focus on the visible and NIR range. In the first step, UV–vis-NIR transmittance measurements were performed on b-D/M structures consisting of an Au film deposited on b-MoO_*x*_ or b-WO_*x*_ as b-D, to evaluate the effect of the different oxides as substrates for the growth of an Au thin film. The UV–vis-NIR transmittance spectra of the b-D/Au structures present a similar profile with a pronounced peak in the visible range, followed by a monotonic decrease toward longer wavelengths (Figure S7). The main peak of b-MoO_*x*_/Au is red-shifted with respect to the b-WO_*x*_/Au sample, and its overall transmission appears higher up to 2450 nm. This indicates that the growth of Au on b-MoO_*x*_ results in a metal layer with enhanced optical transparency compared to the same metal deposited on b-WO_*x*_. Moreover, the electrical conductivity of the b-D/Au bilayers was investigated as well by 4-probe sheet resistance (*R*_sq_) measurements carried out by putting the probes on the Au layer. For the b-MoO_*x*_/Au bilayer, an *R*_sq_ value of 4.4 ± 0.4 Ω/sq was measured, while for b-WO_*x*_/Au, the *R*_sq_ corresponds to 7.9 ± 0.1 Ω/sq, highlighting a lower electrical resistance for b-MoO_*x*_/Au.

The optical and electrical differences between b-MoO_*x*_/Au and b-WO_*x*_/Au bilayers are likely associated with variations in the morphology of the Au film on b-MoO_*x*_ and b-WO_*x*_, strongly influenced by the underlying dielectric material. The dielectric substrate affects the growth of the metallic layer, leading to changes in film continuity, roughness, and effective thickness, all of which impact the metal’s physical properties. The UV–vis-NIR transmittance spectra along with the *R*_sq_ measurements confirm the formation of a more continuous and uniform Au layer on b-MoO_*x*_ with respect to b-WO_*x*_. Therefore, the results obtained by UV–vis-NIR absorption spectra and sheet resistance measurements can be directly related to XPS and AFM characterizations (Section 3.1). The XPS analysis clearly defined the stoichiometry of the dielectric layers on which the Au is grown, which results in Au layers with different optical and electrical features. Such different characteristics of Au on b-MoO_*x*_ and Au on b-WO_*x*_ are directly related to the AFM measurements. The AFM images revealed a more homogeneous and uniform film of Au on b-MoO*_x_*, whereas the Au on b-WO*_x_* is characterized by a higher RMS roughness and by the presence of pores 100 ± 20 nm in diameter. These pores observed for Au on b-WO_*x*_ represent elements of electrical discontinuity in the Au film structure, leading to higher sheet resistance values, while the discontinuous and rough morphology is likely related to additional optical losses leading to a less transparent bilayer.

After analyzing the b-D/Au bilayers, the study was extended to the complete D/M/D architectures including both the chemically symmetric and chemically asymmetric structures. These multilayers were characterized, as for the above studies, by Au and b-D thicknesses of 10 and 6 nm, respectively, while the thickness of the t-D was systematically varied (0–40 nm for t-MoOx; 0–70 nm for t-WOx). In Figure 5, the transmittance spectral changes in the UV–vis-NIR range 350–2450 nm are reported for the chemically symmetric b-MoO*_x_*/Au/t-MoO*_x_* and b-WO*_x_*/Au/t-WO*_x_* multilayers as a function of the top dielectric layer thickness. The upper panels show the absolute transmittance spectra, while the lower panels display the corresponding differential curves calculated by subtracting the spectrum of the respective b-D/Au bilayer (0 nm t-D thickness) from the spectrum of each sample at various t-D thicknesses. For both the chemically symmetric multilayers, some common features were observed in the transmittance spectra upon the deposition of the top dielectric on the b-D/Au substrate. The deposition of the first 10 nm of top dielectric results in an increase in the overall transmittance within the 350–2450 nm spectral range with respect to the corresponding b-D/Au bilayer. Then, a further increase in the top dielectric thickness determines a change of the transmittance maximum and a wavelength red shift, along with a broadening of the transmission band (Figure S3).

**Figure 5:**
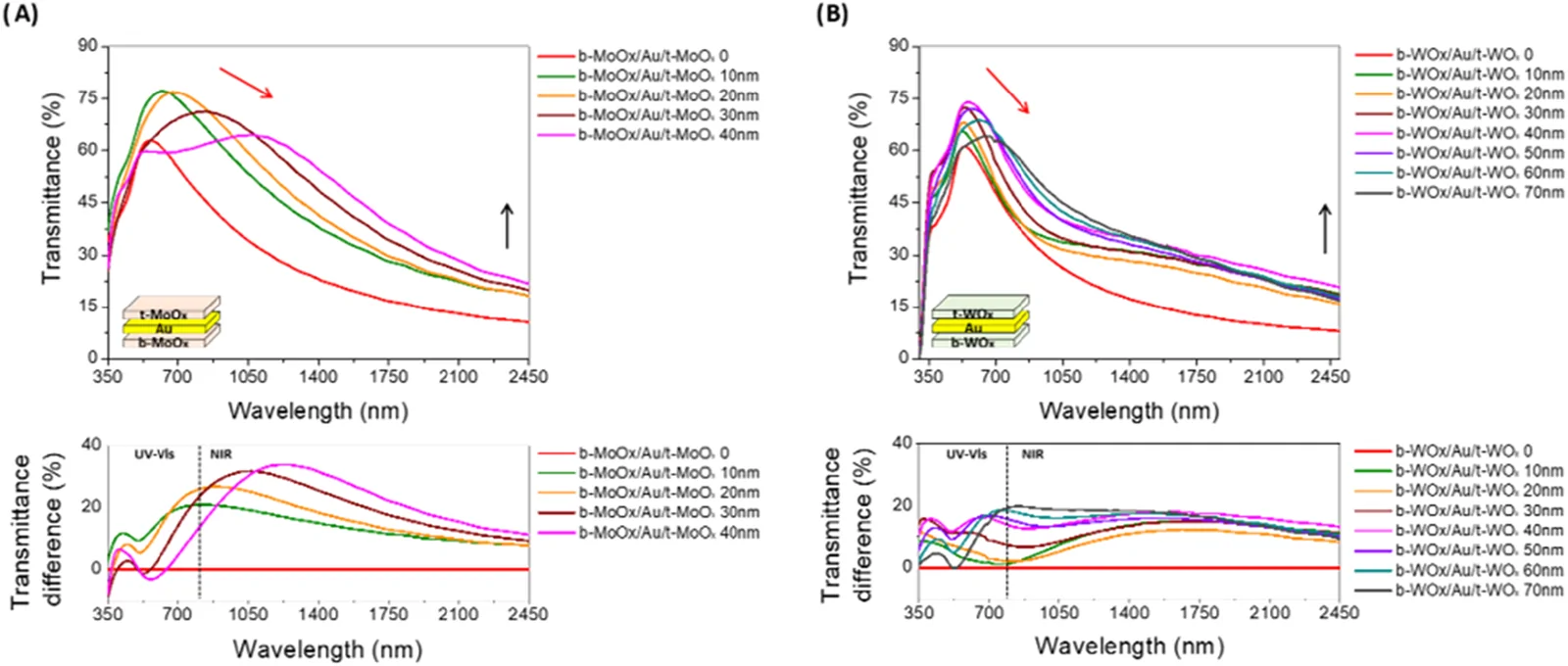
UV–vis-NIR transmittance spectra within the spectral range 350–2450 nm as a function of top dielectric thickness for the chemically symmetric multilayers (A) b-MoO_*x*_/Au/t-MoO_*x*_, and (B) b-WO_*x*_/Au/t-WO_*x*_. For all the multilayers, the thicknesses are 6 nm for b-D, 10 nm for Au, while the thickness of t-D is changed as reported in the labels. For each set of spectra, the corresponding differential curves were calculated by subtracting the spectrum of the respective b-D/Au bilayer (0 nm t-D thickness) from the spectrum of each sample at various t-D thicknesses.

By analyzing the differential transmittance curves, two main optical features emerge in both systems. The first is a negative variation in the UV–vis range, which becomes more pronounced and progressively red-shifted as the thickness of the top dielectric layer increases. The second is an increase in transmittance in the NIR region, also accompanied by a red shift. A noteworthy difference between the two multilayers is that for b-MoO*_x_*/Au/t-MoO*_x_*, the negative variation in the UV–vis extends below zero, indicating that the transmittance becomes lower than that of the starting bilayer for the thicker top dielectric. In contrast, for the b-WO*_x_*/Au/t-WO*_x_* system, the differential transmittance remains always positive, meaning that the addition of the top dielectric layer up to 70 nm thick consistently enhances the absolute transmittance over the entire investigated spectral range compared to the initial bilayer.

Analogously, the spectral changes in transmittance in the UV–vis-NIR range 350–2450 nm are reported in Figure 6 for the chemically asymmetric b-WO*_x_*/Au/t-MoO*_x_* and b-MoO*_x_*/Au/t-WO*_x_* multilayers as a function of the top dielectric layer thickness.

**Figure 6:**
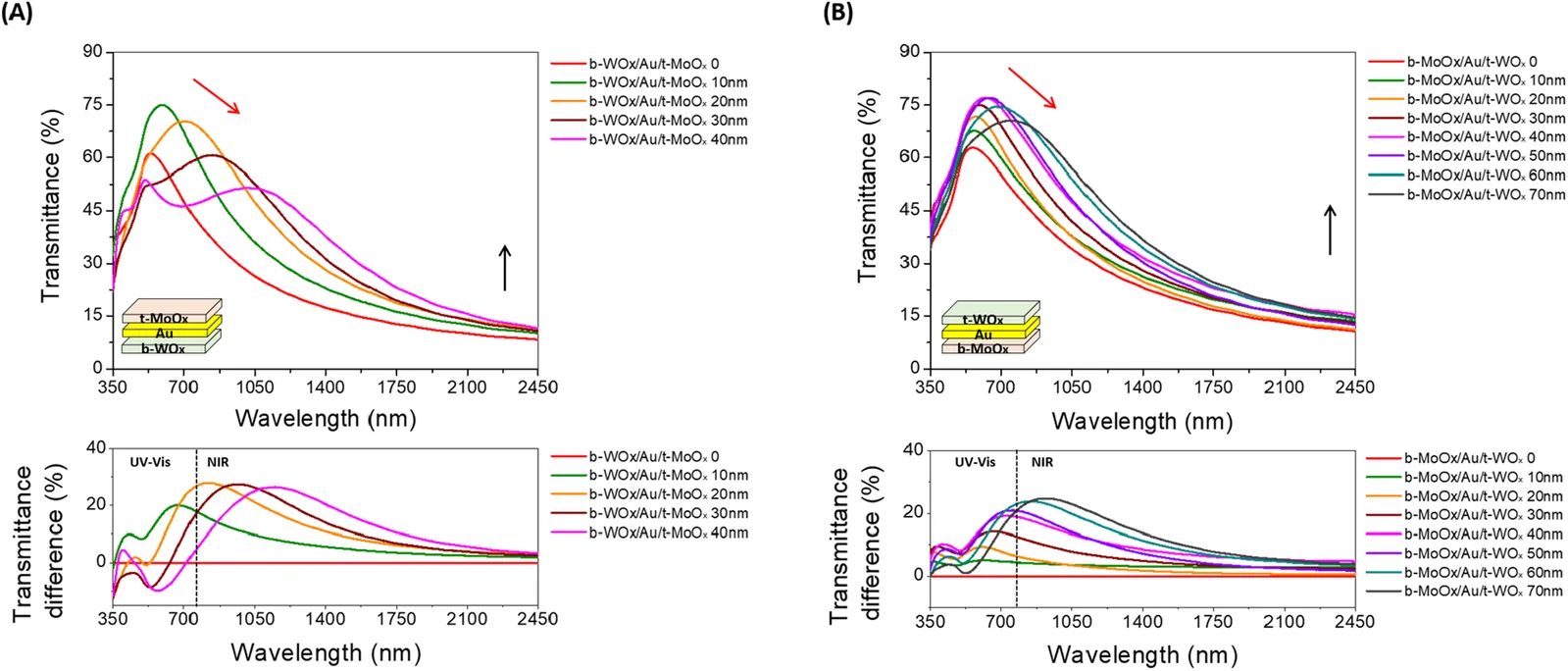
UV–vis-NIR transmittance spectra within the spectral range 350–2450 nm as a function of top dielectric thickness for the chemically asymmetric multilayers (A) b-WO_*x*_/Au/t-MoO_*x*_ and (B) b-MoO_*x*_/Au/t-WO_*x*_. For all the multilayers, the thicknesses are 6 nm for b-D, 10 nm for Au, while the thickness of t-D is changed as reported in the labels. For each set of spectra, the corresponding differential curves were calculated by subtracting the spectrum of the respective b-D/Au bilayer (0 nm t-D thickness) from the spectrum of each sample at various t-D thicknesses.

The spectral variations observed for the asymmetric multilayers are qualitatively analogous to those reported for symmetric systems. An absolute increase in transmittance is observed in the 350–2450 nm spectral range after the deposition of the first dielectric layer 10 nm thick. This is followed by a decrease in transmittance in the UV–vis region and an increase in the NIR region upon increasing the thickness of the top dielectric layer, both accompanied by a red shift (Figure S8).

By comparing the Au-based D/M/D herein investigated, a fundamental optical difference emerges based on the chemical symmetry of the systems. As highlighted by the differential curves, for the case of the symmetric b-D/Au/t-D, modulating the transmittance in the UV–vis range by varying the thickness of the top dielectric layer produces a pronounced increase in transmittance in the overall NIR range as well. In contrast, for the asymmetric multilayers, the UV–vis transmittance is modulated with a more modest variation of the NIR transmittance at the longer wavelength. This feature is evident by comparing, for instance, the value of the transmittance difference at 2200 nm with respect to the corresponding bilayer in the differential curves of the b-D/Au/t-D stacks with a t-D 40 nm thick. For the symmetric b-MoO*_x_*/Au/t-MoO*_x_* and b-WO*_x_*/Au/t-WO*_x_* multilayers, a difference in transmittance of 14% and 12% is calculated, respectively. On the other hand, the differential curves of the asymmetric b-WO*_x_*/Au/t-MoO*_x_* and b-MoO*_x_*/Au/t-WO*_x_* multilayers show a difference of transmittance at 2200 nm with respect to the corresponding bilayer of 6% and 4%, respectively. In this respect, symmetric and asymmetric b-D/Au/t-D architectures provide different strategies to tune the optical response of the electrode. In symmetric b-D/Au/t-D systems, the optical interference conditions allow the transmittance to be maximized simultaneously in both the visible and NIR spectral regions. Conversely, the asymmetric configurations enable a modulation resulting in an enhancement of the transmittance in the visible range, while the transmittance in the lower-energy NIR region is slightly modified and kept less than 20%. This behavior provides an additional degree of freedom for designing transparent electrodes whose spectral selectivity can be exploited to properly tune the multilayers’ transmittance in the UV–vis and NIR range depending on the specific application.

To highlight the spectral selectivity of the Au-based D/M/D herein investigated, the average transmittance values were calculated in the visible and NIR spectral ranges and reported as a function of the top dielectric thickness for applicative purposes (Figure S9). The thickness of the top dielectric can be selected to maximize the optical window in the visible range (400–800 nm), thereby obtaining electrodes with the highest possible transparency for integration into semitransparent PV devices. On the other hand, tuning the transmittance in the first portion of the NIR range of 800–1100 nm is fundamental toward the integration of ST-PSCs with D/M/D electrodes in tandem with silicon solar cells. Finally, for application in smart photovoltaic windows aimed at modulating the heat exchange, the 1100–2500 nm range is also relevant. Focusing on the NIR spectral range, the chemical composition and thickness of the top dielectric layer are observed to play a key role in modulating the NIR transmittance. In particular, the analysis of the first part of the NIR in the range 800–1100 nm showed that the D/M/D systems employing t-MoO*_x_* are characterized by an increase in the NIR transmittance, progressively increasing with t-MoO*_x_* thickness up to approximately 30 nm, then decreasing again. More specifically, for b-MoO*_x_*/Au/t-MoO*_x_*, the NIR transmittance varies from 49% at t-MoO*_x_* 10 nm thick up to 69% at t-MoO*_x_* 30 nm thick. For the case of b-WO*_x_*/Au/t-MoO*_x_*, an analogous behavior is observed, with an NIR transmittance that changes from 31 to 57% as the t-MoO*_x_* is increased from 10 to 30 nm. The D/M/D completed with t-WO*_x_* all show a gradual increase in NIR transmittance with the thickness of the t-WO*_x_* layer. The analysis of the second part of the NIR in the range 1100–2500 nm showed that the D/M/D systems employing t-MoO*_x_* are characterized by a gradual increase in transmittance, while the systems completed with t-WO*_x_* exhibit limited changes in their optical properties. Such spectral tunability is particularly relevant for the integration of these electrodes into ST-PSCs, where the NIR transmission of the top electrode can influence the radiative energy reaching the underlying device layers. When thermal management is required, the NIR transmittance can be reduced through an appropriate selection of the top dielectric material and thickness, thereby limiting the amount of NIR radiation reaching the underlying layers and potentially reducing their thermal load under working conditions. This can be particularly beneficial for mitigating thermally induced degradation and improving the long-term stability of the ST-PSCs. Conversely, higher NIR transmittance can be targeted when transmission of this spectral region is desirable, as in tandem architecture with silicon-based photovoltaics. The control of NIR transmission is also relevant for advanced applications such as BIPV and agrovoltaics, where the optical response of the transparent electrode can be tailored according to the thermal management requirements of the building. Lower NIR transmittance can be advantageous for limiting solar heat gain and excessive heating of indoor environments, whereas higher NIR transmission may be desirable when solar radiation is intentionally exploited for passive heating under colder conditions. Furthermore, a spectral decomposition of the visible range in blue (400–480 nm), green (480–580 nm), and red (580–800 nm) regions highlights an additional degree of tunability in the b-D/Au/t-D multilayers (Figure S10). For b-MoO*_x_*-based systems (Figure S10A,B), the blue component is consistently suppressed, reaching a maximum of 57% for b-MoO*_x_*/Au/t-MoO*_x_*. The green and red regions initially exhibit comparable transmittance of ∼65%, but diverge with increasing top dielectric thickness, resulting in a progressive redistribution of transmitted light toward the red region at the expense of the green. This selective attenuation of high-energy photons may contribute to improved device stability, while the red-enhanced transmission is advantageous for both agrivoltaic applications and tandem integration with silicon bottom cells.

In contrast, b-WO*_x_*-based systems (Figure S10C,D) display a distinct spectral evolution, with an initial maximum in the green region. Increasing the top dielectric thickness leads to a global enhancement of visible transmittance up to a maximum, followed by a gradual decrease.

After analyzing the transmitted light, the reflected component was systematically investigated by means of UV–vis-NIR diffuse reflectance measurements. This aspect is crucial not only for a fundamental understanding of the optical properties of the D/M/D structures but also from a technological perspective, since low reflectance is highly desirable for BIPV and smart window applications, where minimizing reflected light can contribute to ensuring visual comfort and architectural integration. As for the case of the transmittance measurements, the UV–vis-NIR diffuse reflectance spectra were recorded for all the b-D/Au/t-D multilayers as a function of the top dielectric thickness, as shown in Figure 7.

**Figure 7:**
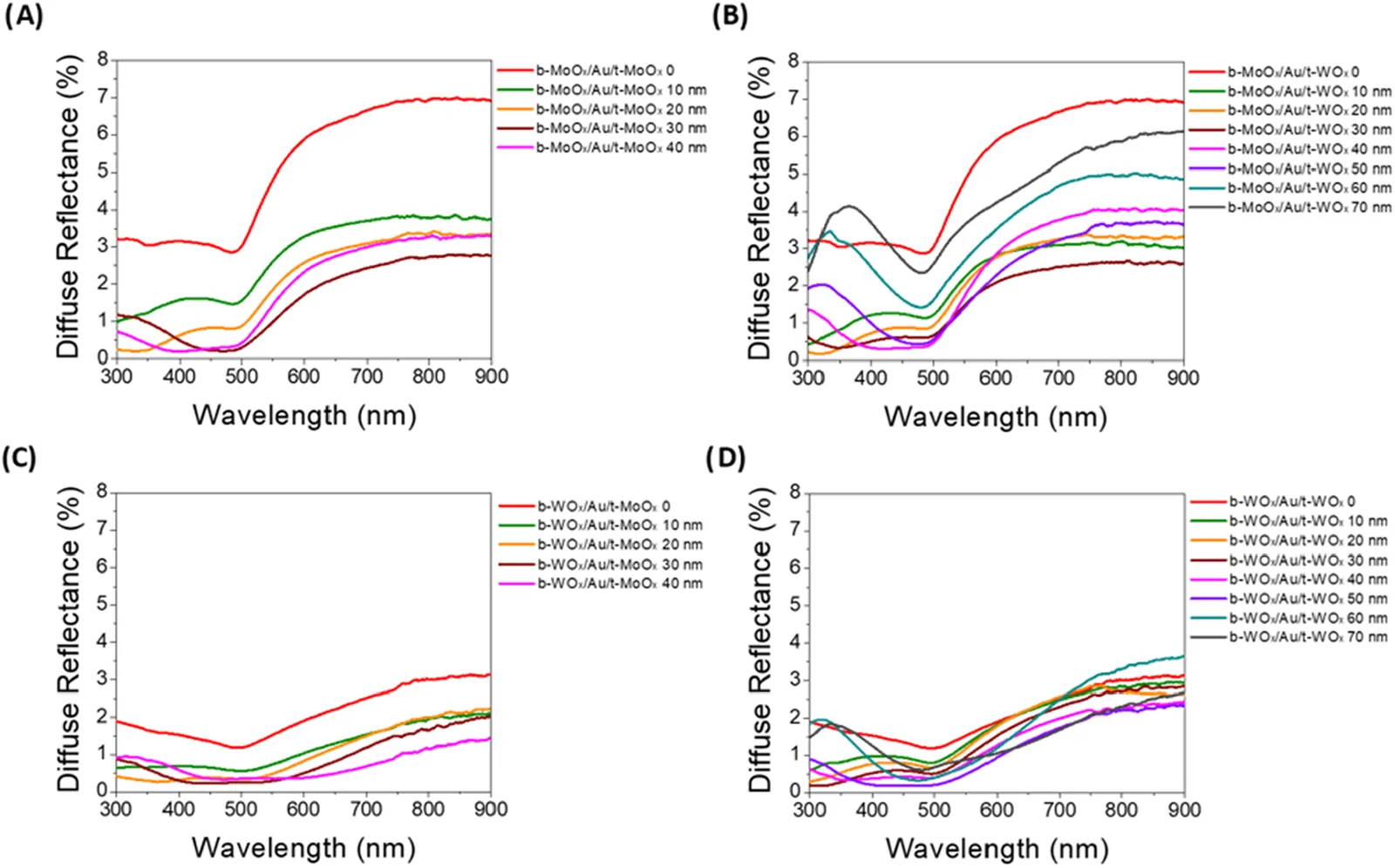
UV–vis-NIR diffuse reflectance spectra within the spectral range 300–900 nm of the b-D/Au/t-D as a function of t-D thickness for (A) b-MoO_*x*_/Au/t-MoO_*x*_, (B) b-MoO_*x*_/Au/t-WO_*x*_, (C) b-WO_*x*_/Au/t-MoO_*x*_, and (D) b-WO_*x*_/Au/t-WO_*x*_. For all the multilayers, the thicknesses are 6 nm for b-D, 10 nm for Au, while the thickness of t-D is changed as reported in the labels.

These measurements reveal that the b-D/Au/t-D presents a UV–vis-NIR diffuse reflectance below 8% for the b-D/Au/t-D with b-MoO_*x*_, and below 4% for the b-D/Au/t-D with b-WO_*x*_, over the measured spectral range. No further relevant changes were observed in the NIR range from 900 nm up to 1800 nm (Figure S11). The morphological differences observed by AFM (Section 3.1) can provide further insights into the distinct optical response of the Au layers deposited on b-MoO_*x*_ and b-WO_*x*_. The Au film grown on b-MoO_*x*_ exhibits a more homogeneous and continuous morphology, characterized by a lower RMS roughness (1.2 nm), whereas the Au layer deposited on b-WO_*x*_ shows a less compact structure with the presence of nanoscale pores 100 ± 20 nm in diameter, and a significantly higher RMS roughness (3.4 nm). These morphological features indicate a different growth mechanism of Au depending on the underlying dielectric layer, which strongly affects the optical response of the metallic film, depending on the chemical composition and stoichiometry monitored by XPS measurements. In particular, the more uniform Au morphology obtained on b-MoO_*x*_ leads to typical behavior for a flat metallic surface characterized by higher optical reflectance (∼8%). Conversely, the discontinuous morphology of Au grown on b-WO_*x*_ introduces local variations in the dielectric environment and nanoscale metal discontinuities, which can promote light trapping and absorption, resulting in a lower diffuse reflectance (<4%). Upon the deposition of the top dielectric on the Au film, a lowering of the diffuse reflectance is observed for all the Au-based D/M/D architectures investigated. However, as the t-WO_*x*_ thickness exceeds 50 nm, an increase in reflectance is observed, likely due to the formation of larger oxide clusters. Low reflectance values are particularly advantageous for the UV–vis range, since minimizing reflected light contributes to maximizing the amount of incident sunlight transmitted to the photoactive layer of the solar cell. Low reflectance also contributes to reduced glare, which improves both aesthetic integration and visual comfort for applications in BIPV and smart windows.^74^

The spectral features in the visible range are strongly affected by both the presence and thickness of the top dielectric layer. In all the investigated b-D/Au/t-D, upon the deposition of the first 10 nm thick dielectric layer, a lowering of the reflectance value is observed. This is in accordance with the corresponding increase in the transmittance for the same samples in the visible range. Further increasing the thickness of t-MoO_*x*_ and t-WO_*x*_ reduces the minimum diffuse reflectance at around 490 nm to nearly 0% at thicknesses of 30 and 50 nm, respectively. The minimum in reflectance is also red-shifted by about 10 nm over this thickness range. At higher top dielectric thicknesses, bimodal spectra are observed, together with an increase in diffuse reflectance. Such changes in the spectral features of the diffuse reflectance can be mainly related to the combination of the interference due to the antireflecting properties of t-MoO_*x*_ and t-WO_*x*_ and the changes of the surface plasmon oscillations sensitive to the dielectric constant of the medium at the interface of the Au layer. Then, the transmittance and diffuse reflectance measurements are coherent but not equivalent, as they probe complementary aspects of the optical response. Their similar spectral trends arise from the interference effects governing the D/M/D multilayers, while transmittance is directly relevant for device applications, and diffuse reflectance provides additional insight into light scattering and optical quality.

For a transparent system to be employed as a top electrode in ST-PSCs, its conductivity must be sufficiently high to allow high charge transport comparable to standard metallic contacts. Therefore, it is essential to evaluate the electrical properties of the b-D/Au/t-D systems prior to their integration into ST-PSCs. To achieve this goal, sheet resistance (*R*_sq_) measurements were performed for the four b-D/Au/t-D architectures investigated in this work as a function of the top dielectric thickness. As reported in Figure 8A, the b-D/Au/t-D systems cluster in two groups depending on the bottom dielectric material. The multilayers fabricated with b-MoO_*x*_ exhibit lower sheet resistance values of 4.2 ± 0.2 Ω/sq, whereas those based on b-WO_*x*_ display comparatively higher sheet resistance of 7.5 ± 0.3 Ω/sq. These data highlight a higher electrical conductivity for multilayers in which Au is deposited on b-MoO_*x*_. This finding is consistent with the UV–vis-NIR spectroscopy results, which suggest a more uniform growth of the Au layer on b-MoO_*x*_ compared to b-WO_*x*_. Furthermore, no significant dependence of the sheet resistance on the top dielectric thickness was observed. This is a positive outcome, indicating that the presence of the nanometric top dielectric layer allows modulation of optical transmittance without adversely affecting the electrical conductivity of the multilayers. The observation that the thickness of the metal oxide capping layer does not affect the overall sheet resistance of its underlying D/M layer has also been reported for other D/M/D systems based on Au and MoO_*x*_.^56^

**Figure 8:**
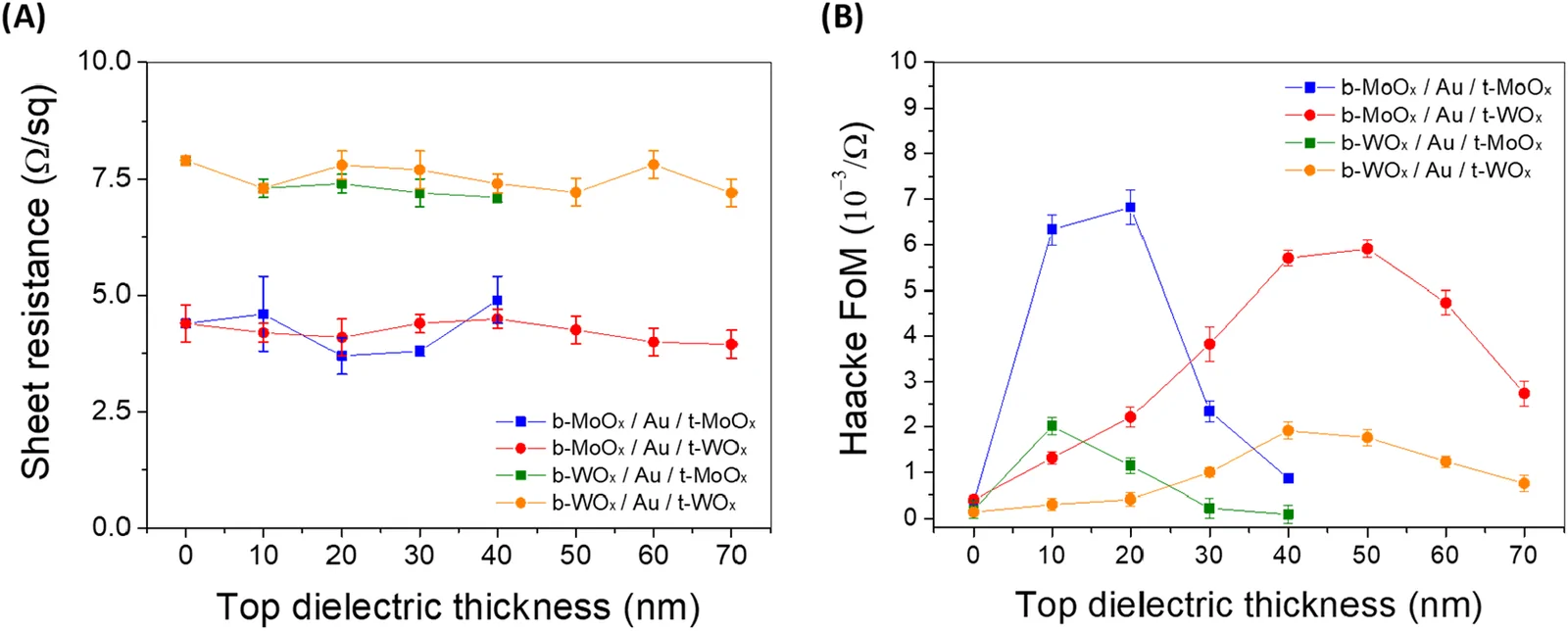
(A) Electrical characterization of b-D/Au/t-D by sheet resistance (*R*_sq_) measurements as a function of top dielectric thickness, providing a quantitative evaluation of electrical conductivity. (B) Haacke figures of merit (Φ_H_) calculated as Φ_H_ = (AT_400–800 nm_)^10^/*R*_sq_, where AT_400–800 nm_ is the average visible transmittance evaluated in the range 400–800 nm as a function of top dielectric thickness. The plot compares b-MoO_*x*_/Au/t-MoO_*x*_ (blue), b-MoO_*x*_/Au/t-WO_*x*_ (red), b-WO_*x*_/Au/t-MoO_*x*_ (green), and b-WO_*x*_/Au/t-WO_*x*_ (orange). All samples feature a 6 nm b-D and a 10 nm Au layer, while the thickness of t-D is changed as reported.

The overall performance of the b-D/Au/t-D transparent electrodes was evaluated by combining their optical properties with their electrical conductivity using the Haacke figure of merit (Haacke FoM, Φ_H_). It is defined as Φ_H_ = T^10^/*R*_sq_, where *T* is the optical transmittance (expressed as a number between 0 and 1) at a specific wavelength or in a specific spectral range, and *R*_sq_ is the sheet resistance of the electrode. The exponent on the transmittance strongly emphasizes the importance of high optical transparency, which is critical for semitransparent devices. The Haacke FoM parameter is widely adopted for transparent electrodes, as it quantitatively balances optical transparency and electrical conductivity.^75,76^ Notably, the Haacke FoM allows the optical and electrical properties of the electrode to be evaluated independently of its integration into the complete photovoltaic device. Therefore, it provides a quantitative assessment of the trade-off between transmittance and sheet resistance at the multilayer level and can be used to identify the most promising multilayer configurations to test as transparent top electrodes prior to their integration in the solar devices. The values of Φ_H_ in the visible range were calculated for the b-D/Au/t-D structures based on the AT_400–800 nm_ and *R*_sq_ values measured, then reported as a function of the thickness of the top dielectric layer in Figure 8B. The data show that both the chemical composition and the thickness of the dielectric layers strongly influence the overall electrode performance. First, the superior properties of b-MoO_X_ as a bottom dielectric for Au film growth, already highlighted by its optical and electrical characteristics with respect to the analogous on b-WO_*x*_, are also reflected in the Φ_H_ values as well, which are higher when the electrode is fabricated using b-MoO*_x_*. Moreover, in all cases, deposition of the top dielectric on Au leads to an increase in Φ_H_ when the thickness is properly tuned, which is a highly favorable feature for applications in semitransparent solar cells. More specifically, for the b-MoO_*x*_/Au/t-MoO_*x*_ electrode, the Φ_H_ initially increases with the dielectric thickness, reaching a maximum of 6.8 × 10^–3^ Ω^–1^ at 20 nm, after which it gradually decreases for thicker layers. In the case of b-MoO_*x*_/Au/t-WO_*x*_ electrodes, the Φ_H_ showed a more gradual increase, peaking at 5.9 × 10^–3^ Ω^–1^ for a thickness of 50 nm, indicating a broader range of thicknesses over which high performance is maintained. b-WO_*x*_/Au/t-MoO_*x*_ configuration exhibited lower overall Φ_H_ values, with a modest peak of 2.1 × 10^–3^ Ω^–1^ at 10 nm and a rapid decrease at higher thicknesses. A similar trend was observed for the symmetric b-WO_*x*_/Au/t-WO_*x*_ electrodes, also showing Φ_H_ with values below 2 × 10^–3^ Ω^–1^ at 40 nm, and a relatively flat trend with a slight lowering at increasing thickness. The Φ_H_ values are crucial for comparing different electrodes, and in this work were used as the benchmark parameters for selecting the b-D/Au/t-D multilayers to be integrated as top electrodes in ST-PSCs.

Therefore, the results indicate that the proper choice of the bottom dielectric plays a crucial role in determining the quality of the Au layer. The Au films deposited on b-MoO_*x*_ exhibit higher uniformity and continuity compared to those grown on b-WO_*x*_, which is consistent with the improved optical transparency and R_sq_ values measured for the former system. At the same time, the choice of the top dielectric layer is equally critical, as it enables fine-tuning of the spectral response of the D/M/D stack across both the visible and near-infrared (NIR) regions. By properly selecting and engineering the top dielectric, it is possible to modulate interference effects and optimize the transmittance and reflectance in targeted wavelength ranges. Overall, these findings highlight that the performance of b-D/Au/t-D systems relies on a synergistic design of both bottom and top dielectric layers; the former governs the morphological and electronic quality of the metal film, while the latter provides an effective handle to tailor the optical response of the multilayer structure.

### 3.3 Integration of b-D/Au/t-D as Top Electrodes in ST-PSCs

For the integration of b-D/Au/t-D systems into ST-PSCs, the multilayer configurations exhibiting the highest Φ_H_ values were selected for each of the four architectures investigated. According to this criterion, the b-D/Au/t-D multilayers with t-MoO_*x*_ 10 nm thick and t-WO_*x*_ 40 nm thick were initially chosen. However, in the latter case, further optimization of the t-WO_*x*_ deposition process was required. In fact, WO_*x*_ is more thermally resistant to sublimation and requires longer deposition times during thermal evaporation to reach a thickness of 40 nm. This correlated with a deterioration in the solar cell performance, as observed in the current–voltage (J-V) characteristics of the solar cell with b-MoO_*x*_/Au/t-WO_*x*_ as the top electrode reported in Figure 9A. The device with a t-WO_*x*_ thickness of 40 nm exhibits a PCE of 7%, which is lower than the 11% for the corresponding device in which the t-WO_*x*_ thickness is reduced to 20 nm. For this reason, a t-WO_*x*_ thickness of 20 nm was selected, as it still allows improved transmittance while mitigating device performance losses. Therefore, the best results obtained with the b-D/Au/t-D herein developed are summarized in Figure 9B. As can be seen, all devices exhibit typical solar cell J-V characteristics, with efficiencies and performance parameters that depend on the specific b-D/Au/t-D electrode architecture.

**Figure 9:**
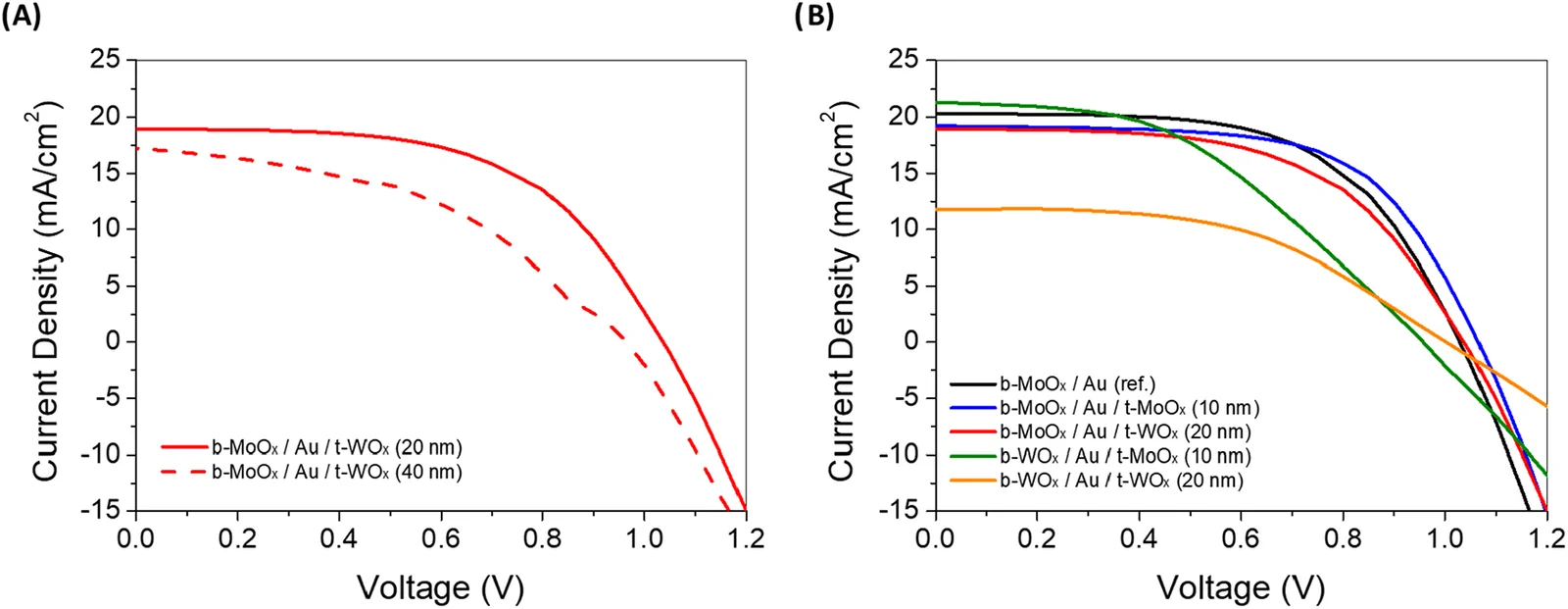
Current density–voltage (J-V) characteristics of semitransparent perovskite solar cells (ST-PSCs) integrated with the optimized b-D/Au/t-D multilayers as top electrodes, in the range 0–1.2 V under 1 Sun illumination. (A) J-V curves for devices using b-MoO_*x*_/Au/t-WO_*x*_ top electrodes with 20 and 40 nm t-WO_*x*_ thickness, illustrating the effect of the top dielectric thickness on the power conversion efficiency. (B) J-V characteristics of ST-PSCs incorporating D/M/D top electrodes selected based on the Haacke figure of merit (FOM) calculated in the visible range for each investigated symmetric and asymmetric D/M/D architectures. For each category of D/M/D electrode, the configuration providing the best balance between optical transparency and D/M/D structural integrity was selected, which corresponds to fixing t-MoO_*x*_ at 10 nm and t-WO_*x*_ at 20 nm for all the D/M/D electrodes. All electrodes feature a 6 nm b-D and a 10 nm Au layer.

The figures of merit of these devices are reported in Table 2. The reference device with a b-MoO_*x*_/Au top electrode with a PCE value of 12% was used as a benchmark for comparison. By introducing an additional 10 nm thick t-MoO_*x*_ layer on top of Au to obtain the symmetric b-MoO_*x*_/Au/t-MoO_*x*_ electrode, a ST-PSC with performance comparable to the reference device was obtained with a PCE of 13%. This demonstrates that the electrode b-MoO_*x*_/Au/t-MoO_*x*_ can be successfully integrated into ST-PSCs without modification of their photovoltaic performance. This approach allows the device efficiency to be preserved while improving the transparency in the visible spectral range. In the case where the outermost layer is t-WO_*x*_, as mentioned above, a thickness of 40 nm is associated with the highest average transmittance in the visible range but also with a marked deterioration in device performance, likely related to a worsening of interfacial quality during the longer deposition process. On the basis of these results, an optimization of the t-WO_*x*_ thickness was carried out, identifying 20 nm as a compromise between functionality and minimized thermal damage. Reducing the t-WO_*x*_ thickness to 20 nm helped limit PCE losses, resulting in a PCE of 11%, comparable to that of the reference device. On the other hand, when the top electrode is designed with b-WO_*x*_, typically lower figures of merit were observed for the resulting devices. In fact, the top electrodes b-WO_*x*_/Au/t-WO_*x*_ and b-WO_*x*_/Au/t-MoO_*x*_ exhibit overall lower efficiencies compared with their b-MoO_*x*_ counterparts. This aspect agrees with the UV–vis-NIR spectroscopy and R_sq_ measurements, which suggested a better and more homogeneous growth of the Au layer on b-MoO_*x*_ compared to that on b-WO_*x*_. No photovoltaic performance was detected for the system b-WO_*x*_/Au/t-WO_*x*_ with a t-WO_*x*_ thickness of 40 nm.

**Table 2:** Figures of Merit of ST-PSCs (Short-Circuit Current, *J*_sc_; Open-Circuit Voltage, *V*_oc_; Fill Factor, FF; Series Resistance, *R*_s_; Shunt Resistance, *R*_shunt_; Haacke FoM, Φ_H_; Power Conversion Efficiency, PCE) for Devices with Different b-D/Au/t-D Top Electrodes, Measured under Standard (1 Sun) Illumination from the FTO Side^a^

b-D/M/t-D electrode	*J*_sc_ (mA/cm^2^)	*V*_oc_ (V)	FF (%)	*R*_s_ (Ω cm^2^)	*R*_shunt_ (kΩ cm^2^)	PCE (%)	Φ_H_ (10^–3^ Ω^–1^)
b-MoO*_x_*/Au (standard)	20.3 ± 0.5	1.03 ± 0.02	59 ± 1	11.34	5.09	12 ± 1	0.36
b-MoO*_x_*/Au/t-MoO*_x_* (10 nm)	19.2 ± 0.8	1.07 ± 0.04	62 ± 2	12.47	2.17	13 ± 1	6.33
b-MoO*_x_*/Au/t-WO*_x_* (20 nm)	18.9 ± 0.6	1.04 ± 0.04	56 ± 3	14.09	2.73	11 ± 1	2.22
b-MoO*_x_*/Au/t-WO*_x_* (40 nm)	17.2 ± 0.5	0.97 ± 0.03	44 ± 2	15.45	0.27	7 ± 2	5.67
b-WO*_x_*/Au/t-MoO*_x_* (10 nm)	21.3 ± 0.8	0.96 ± 0.04	44 ± 3	21.34	0.76	9 ± 1	2.02
b-WO*_x_*/Au/t-WO*_x_* (20 nm)	11.8 ± 0.7	1.00 ± 0.02	51 ± 1	35.15	1.42	6 ± 1	0.41
b-WO*_x_*/Au/t-WO*_x_* (40 nm)							1.92

a All the ST-PSCs have a b-D/Au/t-D top electrode following the structure b-D (6 nm)/Au (10 nm)/t-D (thickness as indicated).

Therefore, the performance of the herein reported b-D/Au/t-D multilayers tested as top electrodes in ST-PSCs is in line with recent results obtained for ST-PSCs using various D/M/D transparent top electrodes. The PCE of 13 ± 1% achieved with the symmetric b-MoO*_x_*/Au/t-MoO*_x_* architecture matches values typically reported in the literature for optimized Au-based D/M/D electrodes.^50^ In comparison to Ag-based systems, which can also reach similar efficiencies, the use of Au as the metallic core overcomes limited reliability due to its higher resistance to oxidation^77^ and lower reactivity with halides from the perovskite layer typical of Ag thin films.^52^ Furthermore, this study introduces a significant advancement in spectral selectivity. While most literature focuses on achieving maximum visible or NIR transparency, in this work the Au-based D/M/D chemically symmetric and asymmetric architectures were deeply investigated to provide a clear overview on the effect of the chemical composition and thickness of each layer involved in the fabrication of the chemically symmetric and asymmetric stack, and reveal a level of spectral control that is rarely addressed in standard D/M/D studies.

Concerning the Haacke FoM values of the D/M/D multilayers tested as the top electrode for ST-PSCs, the comparison with the PCE values highlights that a higher Haacke FoM does not necessarily translate into improved PCE, as the actual device photovoltaic performance depends on the whole device architecture and interfacial processes. For instance, the standard ST-PSC exhibits a relatively low Haacke FoM due to its opaque top electrode while showing a high PCE. Similarly, for ST-PSCs employing b-MoO_*x*_/Au/t-WO_*x*_ as the top electrode, increasing the t-WO_*x*_ thickness from 20 to 40 nm resulted in an increase in the Haacke FoM up to 5.67, but with a deterioration of the device performance, likely due to structural modifications induced upon integration of the D/M/D electrode into the device. Thus, the Haacke FoM can guide the selection of the most suitable D/M/D multilayers to integrate in solar cells and in evaluating their spectral selectivity in specific spectral ranges, and complementarily, the PCE values demonstrate the actual performance of the obtained device.

Finally, to better rationalize the observed PCE values, the series resistance (*R*_s_) and shunt resistance (*R*_shunt_) of the tested devices were extracted from the J-V characteristics. These parameters were determined from the slopes of the linear regions of the J-V curve near the open-circuit voltage (*V*_oc_) and short-circuit current (*J*_sc_), respectively. The series resistance (*R*_s_) was estimated from the slope of the J-V curve in the high-voltage region close to *V*_oc_, where resistive losses associated with charge transport through the electrodes, interfaces, and active layers dominate the device response. Conversely, the shunt resistance (*R*_shunt_) was obtained from the slope of the J-V curve near *J*_sc_, where leakage pathways and parasitic currents are more pronounced if present. The analysis of these parameters provides valuable insight into both the device architecture and the quality of the fabrication process. *R*_s_ reflects the effectiveness of the material design and the charge transport properties within the device. For optimal photovoltaic performance, *R*_s_ should be as low as possible, since large values hinder current flow, thereby reducing the fill factor and ultimately the device efficiency. On the other hand, *R*_shunt_ is mainly associated with leakage currents caused by structural defects, pinholes, or imperfect interfaces generated during the fabrication process. Therefore, high *R*_sh_ values are desirable, as they indicate minimal parasitic current losses and a reduced density of fabrication-related defects. In the devices investigated here, the extracted *R*_s_ and *R*_shunt_ values differ from those of the reference device, suggesting that the adopted electrode configuration affects the electrical characteristics of the solar cells. With respect to the standard cell, the ST-PSCs with b-MoO_*x*_/Au/t-MoO_*x*_ as top electrode show a slight increase in *R*_s_, suggesting a somewhat higher, yet still acceptable, resistance to charge transport through the device. A similar behavior is observed for the device integrated with b-WO_*x*_/Au/t-MoO_*x*_, in which the top dielectric is 10 nm thick. As the t-MoO_*x*_ is increased up to 40 nm, *R*_s_ increases and *R*_shunt_ decreases, reflecting a lowering in charge transport and the presence of shunt paths leading to electrical losses. Analogously, for the devices with b-WO_*x*_, *R*_s_ and *R*_shunt_ reflect changes in resistance values which result in inferior performance compared to the device fabricated on b-MoO_*x*_.

In conclusion, the highest device performance is achieved with electrodes employing b-MoO_*x*_ as the first layer of the electrode stack, yielding a PCE comparable to that of the standard cell. Comparable performances between D/M/D-based devices and standard solar cells have been reported for many optimized D/M/D systems integrated in third-generation solar cells.^49^

## 4 Conclusions

In this work, Au-based D/M/D multilayers were investigated in both chemically symmetric and asymmetric configurations to develop high-performance transparent top electrodes for ST-PSCs. The Au-based D/M/D architectures were deeply investigated to provide a clear overview of the effect of the chemical composition, morphology, and thickness of each layer of the D/M/D stacks and revealed a high level of optical control. The symmetric D/M/D configurations tend to modify the transmittance in the visible and NIR ranges simultaneously, while the asymmetric D/M/D configurations enable the independent modulation of visible and NIR spectral ranges. This independent tunability is particularly valuable for designing multifunctional electrodes that are not only transparent but also spectrally selective, modulating the NIR transmission to reduce device overheating while maintaining a high visible transparency.

In addition, the D/M/D multilayers were carefully optimized to maintain high electrical conductivity and successfully integrated as transparent top electrodes in ST-PSCs. The performance of the ST-PSCs provided with Au-based D/M/D multilayers was in line with recent results obtained for ST-PSCs using various D/M/D transparent top electrodes. The PCE of 13 ± 1% achieved with the symmetric b-MoO_*x*_/Au/t-MoO_*x*_ architecture matches the benchmark values reported in the literature for optimized Au-based D/M/D electrodes. In comparison to Ag-based systems, which can also reach similar efficiencies, the use of Au as the metallic core provides significantly better long-term reliability due to its higher resistance to oxidation and lower reactivity with halides from the perovskite layer. In conclusion, the spectral selectivity and high electrical conductivity make asymmetric D/M/D multilayers an alternative platform for next-generation ST-PSCs, with potential applications spanning building-integrated photovoltaics, agrivoltaics, tandem solar cells, and smart windows; it may allow for achieving better solar cell stability and solar heat management.

## Supplementary Material



## Notes

Additional structural, morphological, and optical characterizations of the symmetric and asymmetric D/M/D multilayers; AFM to determine RMS surface roughness, while cross-sectional SEM and EDS to analyze bulk morphology and interfacial composition; optical and electrical features of the bottom bilayers evaluated through UV–vis-NIR transmittance and sheet resistance measurements; transmittance maxima, wavelength shifts, and band-averaged transmittance across visible, tandem, and thermal NIR ranges as a function of top dielectric thickness; and color-decomposed visible transmittance and UV–vis-NIR diffuse reflectance spectra to complete the optical characterization (PDF)
