# Supplementary material for: Symmetric and Asymmetric Dielectric/Metal/Dielectric Electrodes for Semitransparent Perovskite Solar Cells
Source: ACS Omega. 2026 Sep 29;11(40):61613–30. doi: 10.1021/acsomega.6c07322 (PMC13670286; doi:10.1021/acsomega.6c07322)
Supplement: Supplementary file 1 [file ao6c07322_si_001.pdf]

SUPPORTING INFORMATION

Symmetric and Asymmetric Dielectric/Metal/Dielectric Electrodes for Semi-Transparent Perovskite Solar Cells

Vittorio Ferrara[1], Silvia Orecchio[1], Giuseppe Arrabito[1], Fabio Principato[1], Alessandro Auditore[2], Valentina Spampinato[2], Antonino Licciardello[2], Valeria Vetri[1], Michelangelo Scopelliti[1,], Bruno Pignataro[1,*]*

[1] Department of Physics and Chemistry - Emilio Segrè, University of Palermo, viale delle Scienze, Palermo 90128, Italy

[2] Department of Chemical Sciences, University of Catania, viale A. Doria 6, 95125 Catania, Italy

* Corresponding authors: Bruno Pignataro (bruno.pignataro@unipa.it), Michelangelo Scopelliti (michelangelo.scopelliti@unipa.it)

Table of contents:

Top-view SEM images of the Au film on b-MoO_x and b-WO_x ………..………….. S1

Roughness values from AFM images of the b-D/Au/t-D ………………………….…. TS1

Cross-sectional SEM images of the b-D/Au/t-D …………………………………… S2

EDS analysis of the b-MoO_x/Au/t-MoO_x multilayer …………………………………. S3

EDS analysis of the b-MoO_x/Au/t-WO_x multilayer ……………………..…………. S4

EDS analysis of the b-WO_x/Au/t-MoO_x multilayer …………………………..………. S5

EDS analysis of the b-WO_x/Au/t-WO_x multilayer ……………………………….…. S6

UV-Vis-NIR transmittance spectra of b-MoO_x/Au and b-WO_x/Au and R_{sq} ………. S7

Transmittance maximum and wavelength shifts for b-D/Au/t-D multilayers……… S8

Average transmittance values as function of the top dielectric thickness ………..... S9

Average Transmittance in blue, green and red spectral ranges….………………..... S10

UV-Vis-NIR reflectance spectra within 300–1800 nm of the b-D/Au/t-D ………… S11

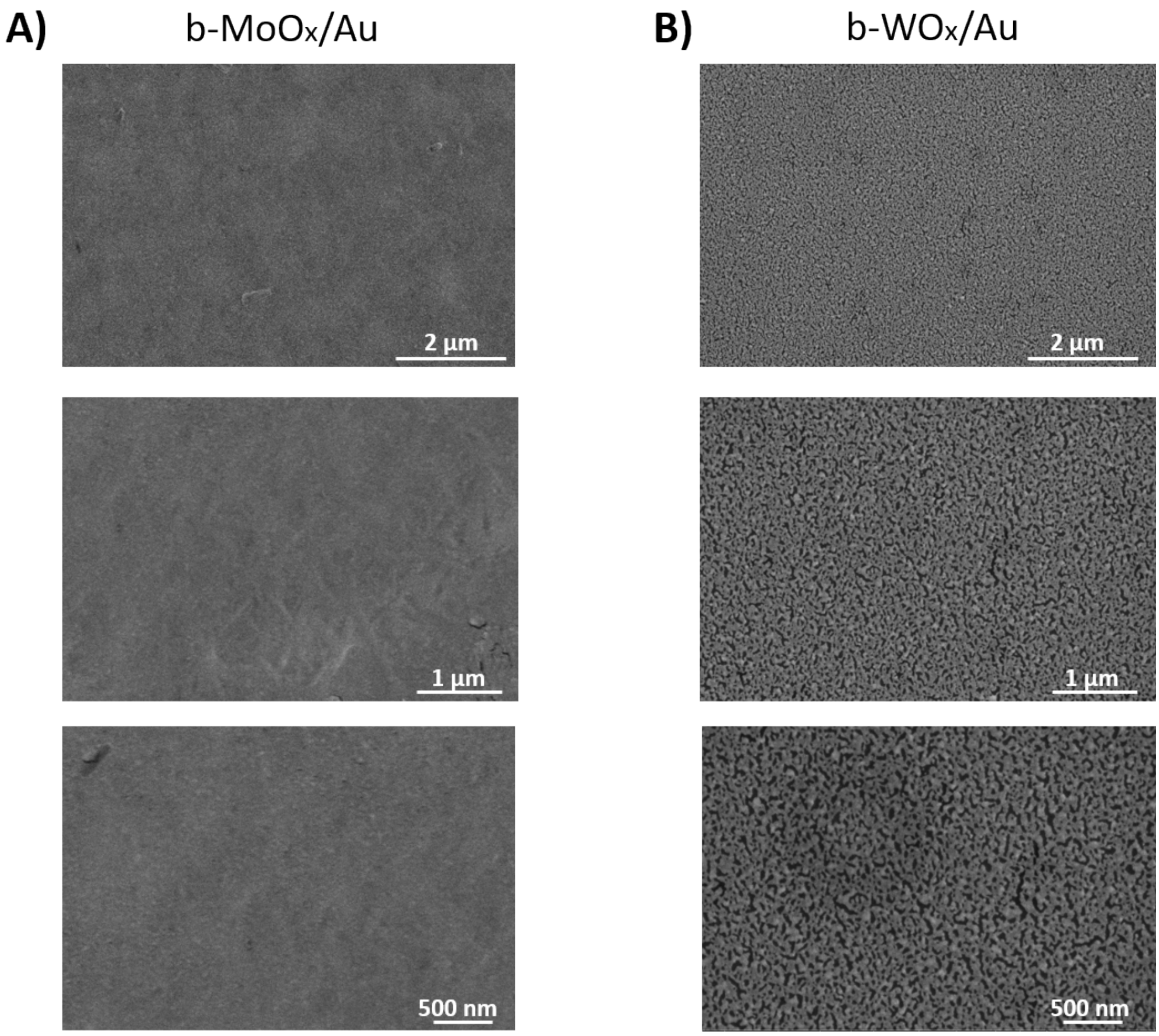

Figure S1. Top-view scanning electron microscopy (SEM) images of the Au film of nominal thickness 10 nm deposited A) on b-MoO_x 6 nm thick, and B) on b-WO_x 6 nm thick, at different magnifications. The images show the homogeneous surface morphology of b-MoO_x/Au, and the presence of Au clusters and pores for b-WO_x/Au.

Table TS1. Root mean square (RMS) roughness values obtained by 2 µm × 2 µm AFM 3D-images of the b-D/Au/t-D.

Sample	RMS Roughness (nm)
glass substrate	1.0
b-MoO_x	1.2
b-WO_x	1.0
b-MoO_x/Au	1.2
b-WO_x/Au	3.4
b-MoO_x/Au/t-MoO_x	2.3
b-MoO_x/Au/t-WO_x	1.4
b-WO_x/Au/t-MoO_x	1.5
b-WO_x/Au/t-WO_x	1.4

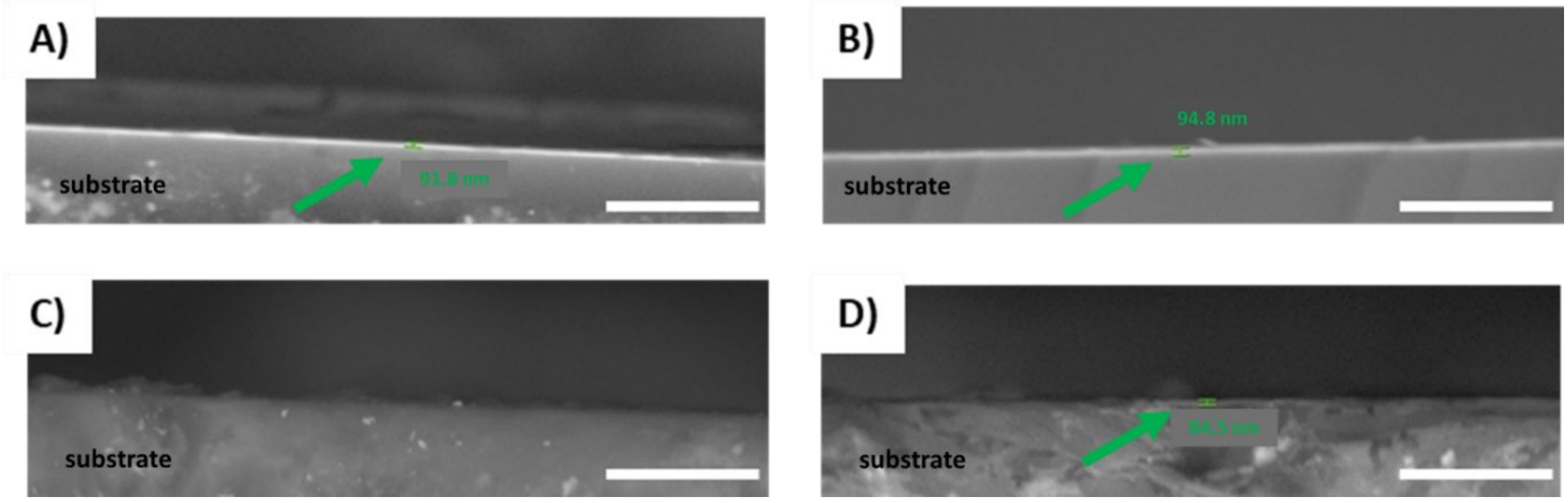

Figure S2. Cross-sectional SEM images of A) b-MoO_x/Au/t-MoO_x, B) b-MoO_x/Au/t-WO_x, C) b-WO_x/Au/t-MoO_x, and D) b-WO_x/Au/t-WO_x. The b-D thickness is 6 nm and Au is 10 nm for all the samples, while t-D is 40 nm for t-MoO_x and 70 nm for t-WO_x. The green arrows indicate the b-D/Au/t-D multilayer, with representative thickness measurements indicating an estimated thickness below 100 nm. For C) b-WO_x/Au/t-MoO_x the presence of the layer is not clearly evident in these acquisition conditions. The images were acquired at 18000× magnification, and scale bars are 3 μm.

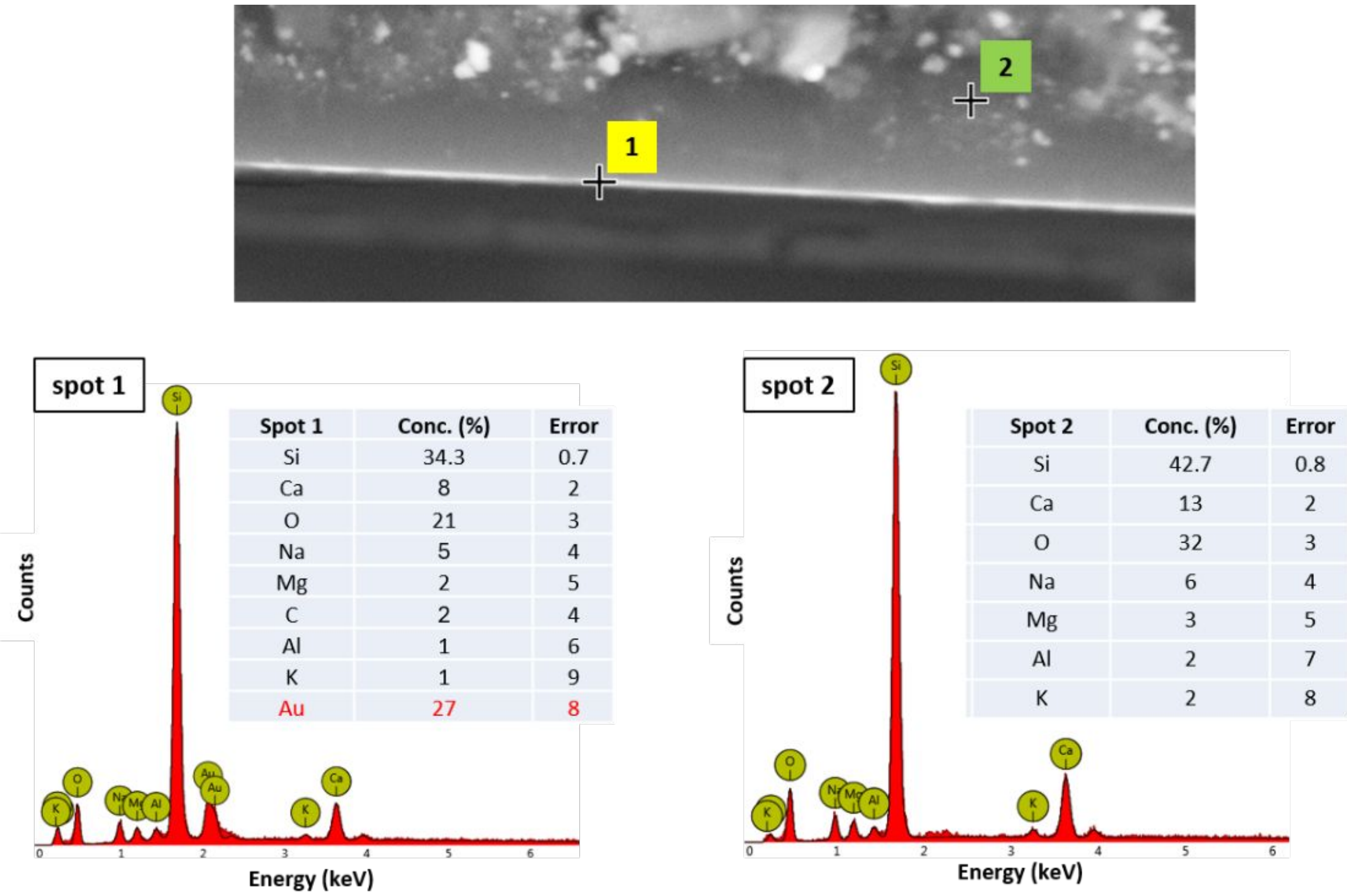

Spot 1	Conc. (%)	Error
Si	34.3	0.7
Ca	8	2
O	21	3
Na	5	4
Mg	2	5
C	2	4
Al	1	6
K	1	9
Au	27	8

Spot 2	Conc. (%)	Error
Si	42.7	0.8
Ca	13	2
O	32	3
Na	6	4
Mg	3	5
Al	2	7
K	2	8

Figure S3. Cross-sectional SEM image and EDS analysis of the b-MoO_x/Au/t-MoO_x multilayer. The EDX spectra were collected on the surface (spot 1, yellow) and on the bulk of the glass substrate (spot 2, green) as control. The elemental composition obtained from each analyzed spot is reported in the corresponding tables. The data refers to D/M/D consisting of b-D layers 6 nm thick, Au layer 10 nm thick and t-D layer 40 nm thick.

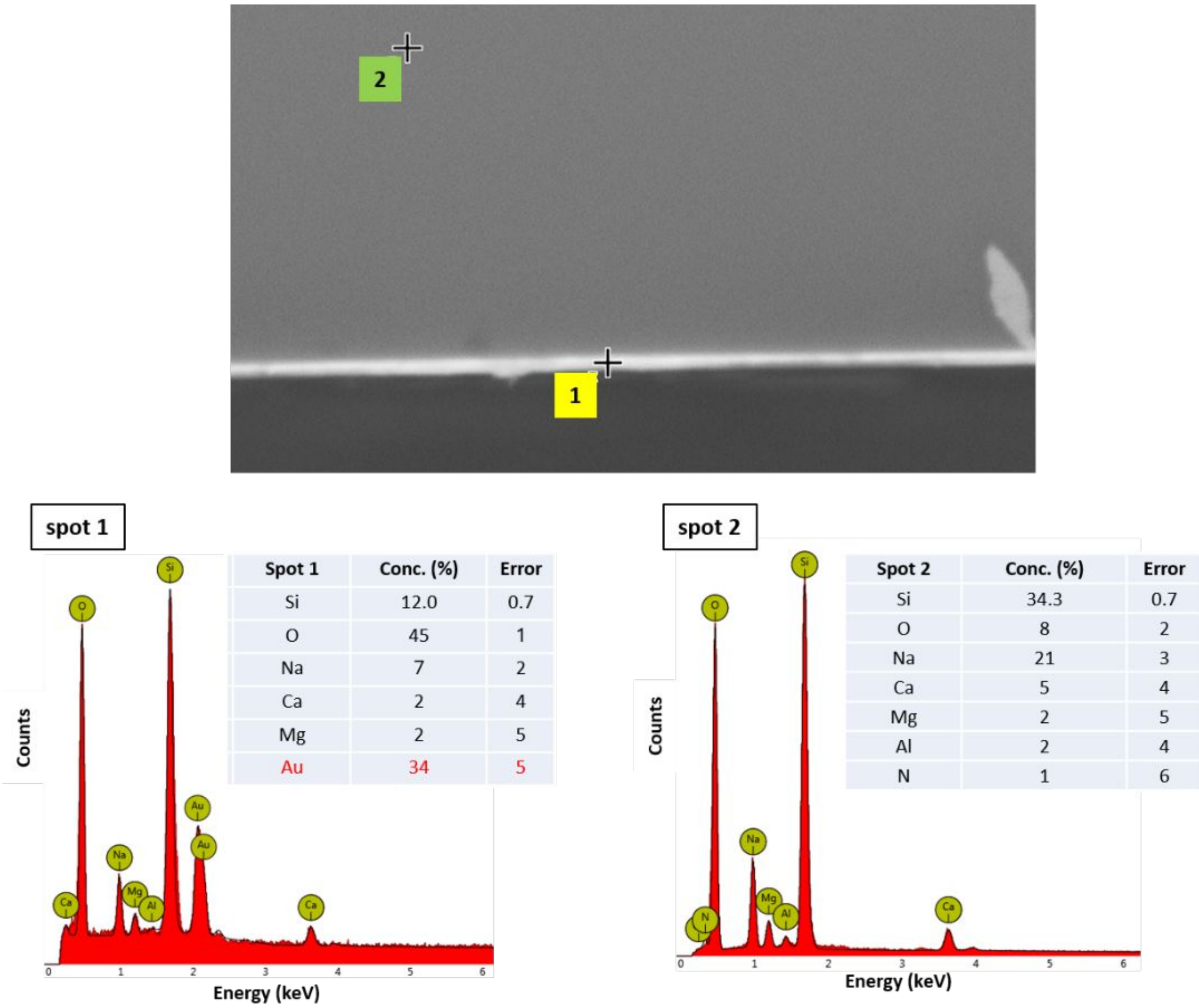

Spot 1	Conc. (%)	Error
Si	12.0	0.7
O	45	1
Na	7	2
Ca	2	4
Mg	2	5
Au	34	5

Spot 2	Conc. (%)	Error
Si	34.3	0.7
O	8	2
Na	21	3
Ca	5	4
Mg	2	5
Al	2	4
N	1	6

Figure S4. Cross-sectional SEM image and EDS analysis of the b-MoO_x/Au/t-WO_x multilayer. The EDX spectra were collected on the surface (spot 1, yellow) and on the bulk of the glass substrate (spot 2, green) as control. The elemental composition obtained from each analyzed spot is reported in the corresponding tables. The data refers to D/M/D consisting of b-D layers 6 nm thick, Au layer 10 nm thick and t-D layer 40 nm thick.

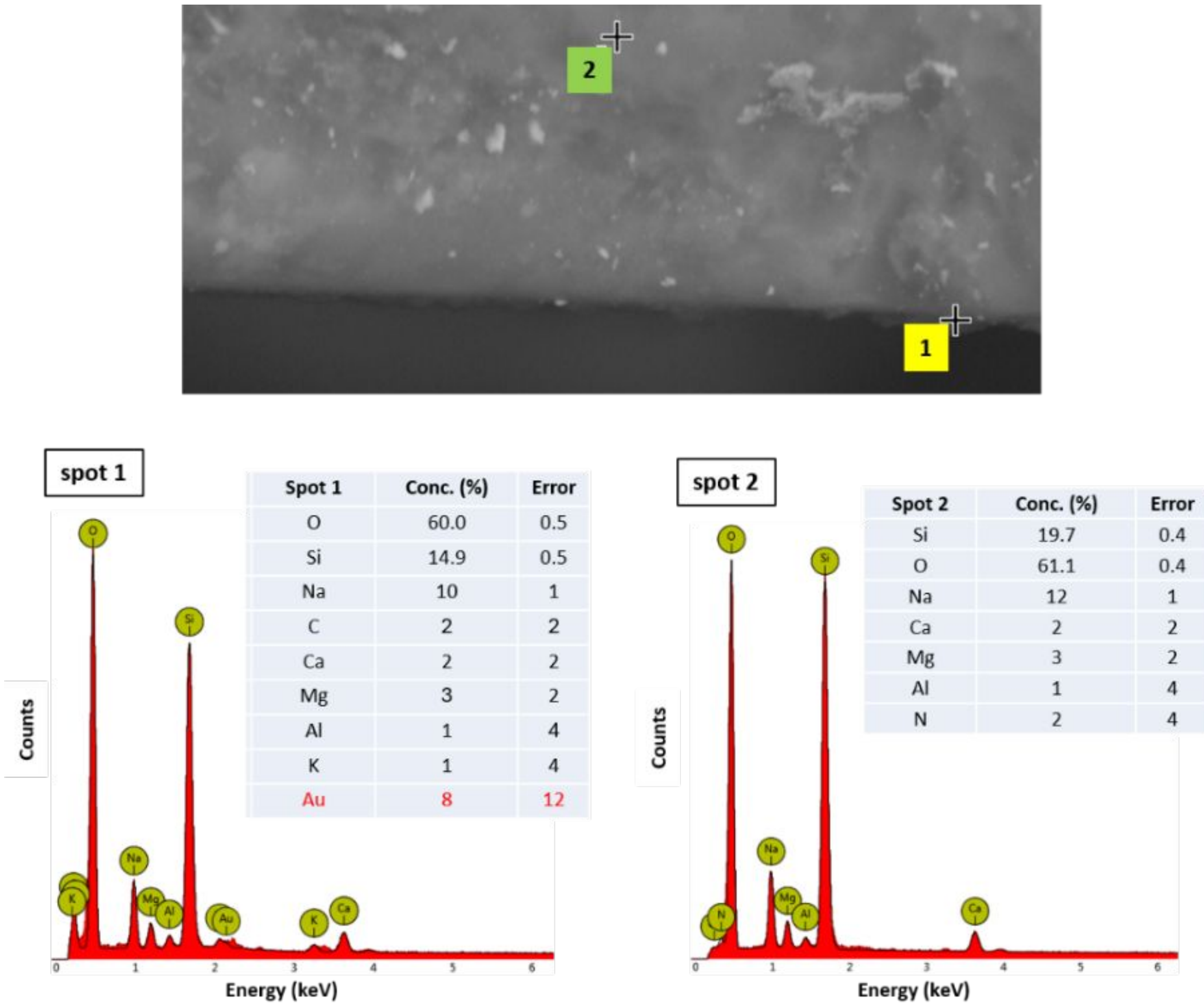

Spot 1	Conc. (%)	Error
O	60.0	0.5
Si	14.9	0.5
Na	10	1
C	2	2
Ca	2	2
Mg	3	2
Al	1	4
K	1	4
Au	8	12

Spot 2	Conc. (%)	Error
Si	19.7	0.4
O	61.1	0.4
Na	12	1
Ca	2	2
Mg	3	2
Al	1	4
N	2	4

Figure S5. Cross-sectional SEM image and EDS analysis of the b-WO_x/Au/t-MoO_x multilayer. The EDX spectra were collected on the surface (spot 1, yellow) and on the bulk of the glass substrate (spot 2, green) as control. The elemental composition obtained from each analyzed spot is reported in the corresponding tables. The data refer to D/M/D consisting of b-D layers 6 nm thick, Au layer 10 nm thick and t-D layer 40 nm thick.

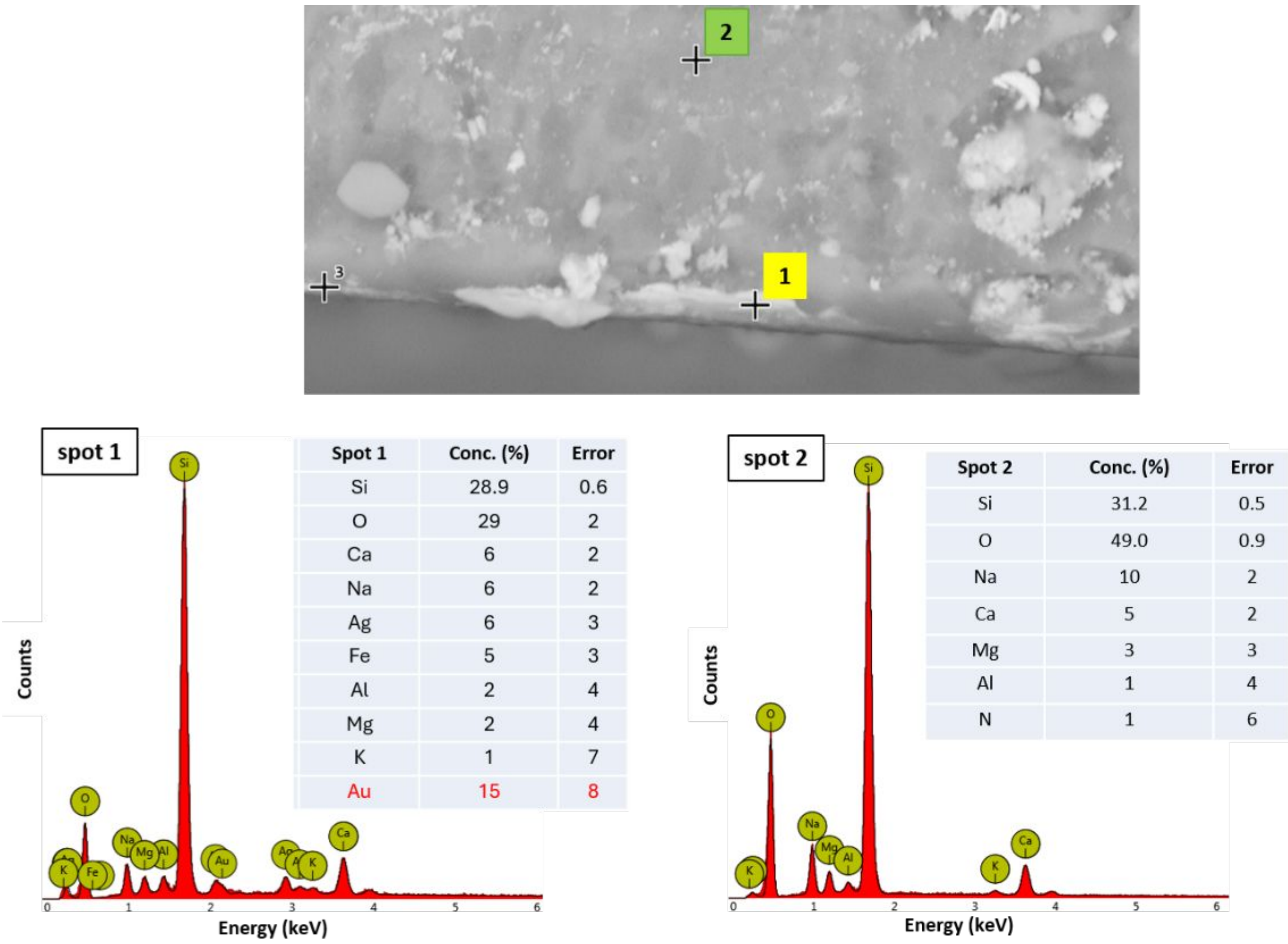

Spot 1	Conc. (%)	Error
Si	28.9	0.6
O	29	2
Ca	6	2
Na	6	2
Ag	6	3
Fe	5	3
Al	2	4
Mg	2	4
K	1	7
Au	15	8

Spot 2	Conc. (%)	Error
Si	31.2	0.5
O	49.0	0.9
Na	10	2
Ca	5	2
Mg	3	3
Al	1	4
N	1	6

Figure S6. Cross-sectional SEM image and EDS analysis of the b-WO_x/Au/t-WO_x multilayer. The EDX spectra were collected on the surface (spot 1, yellow) and on the bulk of the glass substrate (spot 2, green) as control. The elemental composition obtained from each analyzed spot is reported in the corresponding tables. The data refer to D/M/D consisting of b-D layers 6 nm thick, Au layer 10 nm thick and t-D layer 40 nm thick.

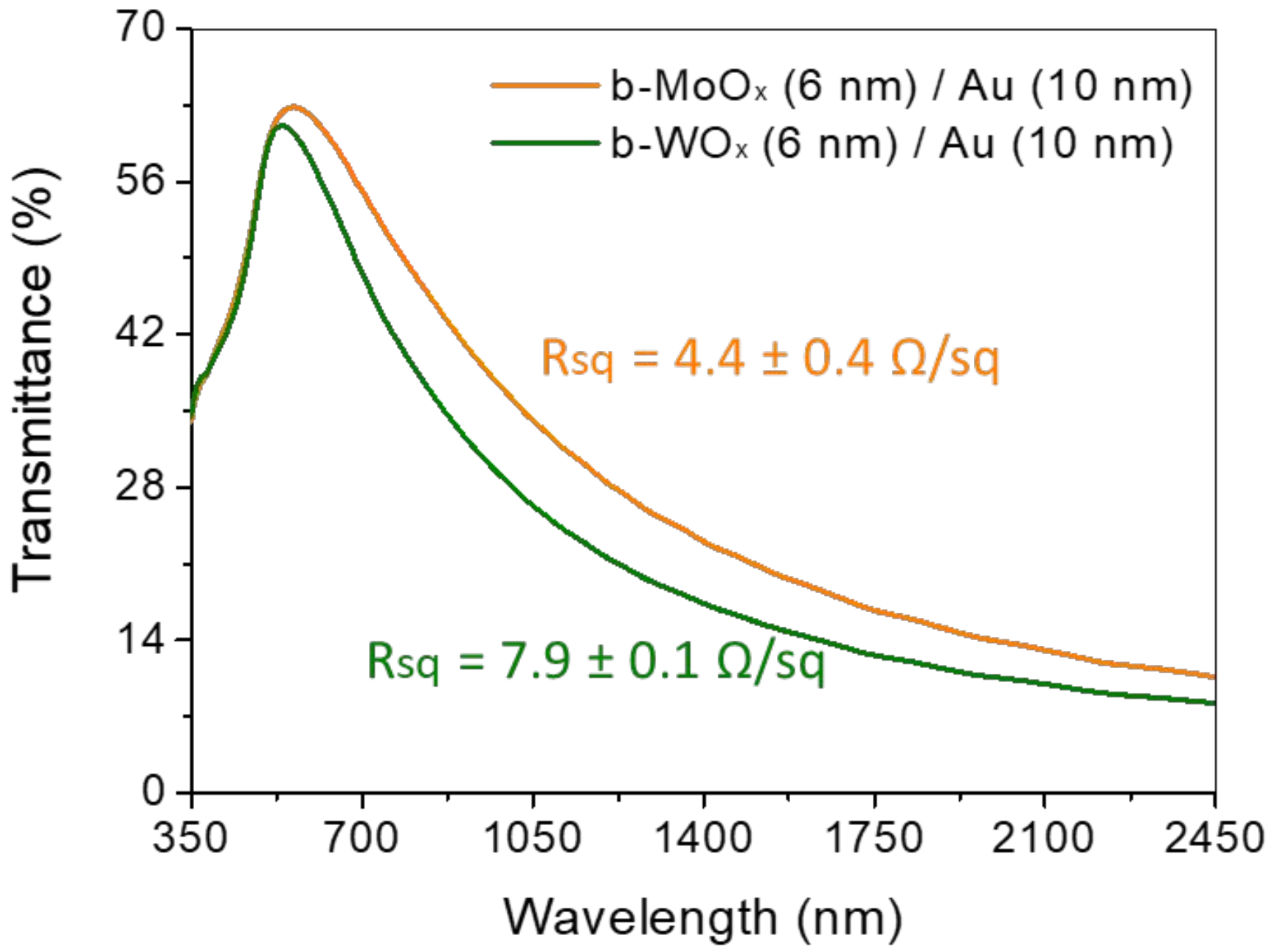

Figure S7. UV-Vis-NIR transmittance spectra in the range 350–2450 nm of b-MoO_x/Au (orange) and b-WO_x/Au (green) and the corresponding values of sheet resistance (R_{sq}).

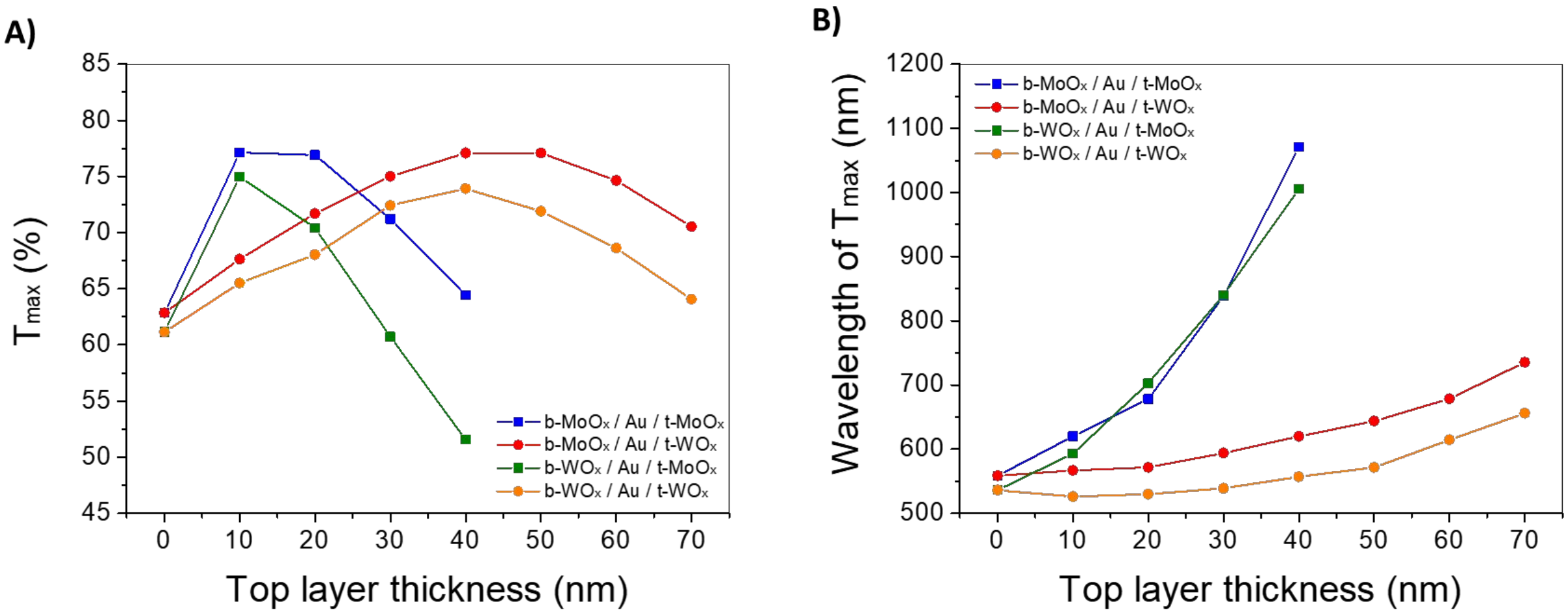

Figure S8. A) Transmittance maximum and B) wavelength shifts for b-D/Au/t-D multilayers b-MoO_x/Au/t-MoO_x (blue), b-MoO_x/Au/t-WO_x (red), b-WO_x/Au/t-MoO_x (green), and b-WO_x/Au/t-WO_x (orange). The b-D thickness is 6 nm and Au is 10 nm for all the samples.

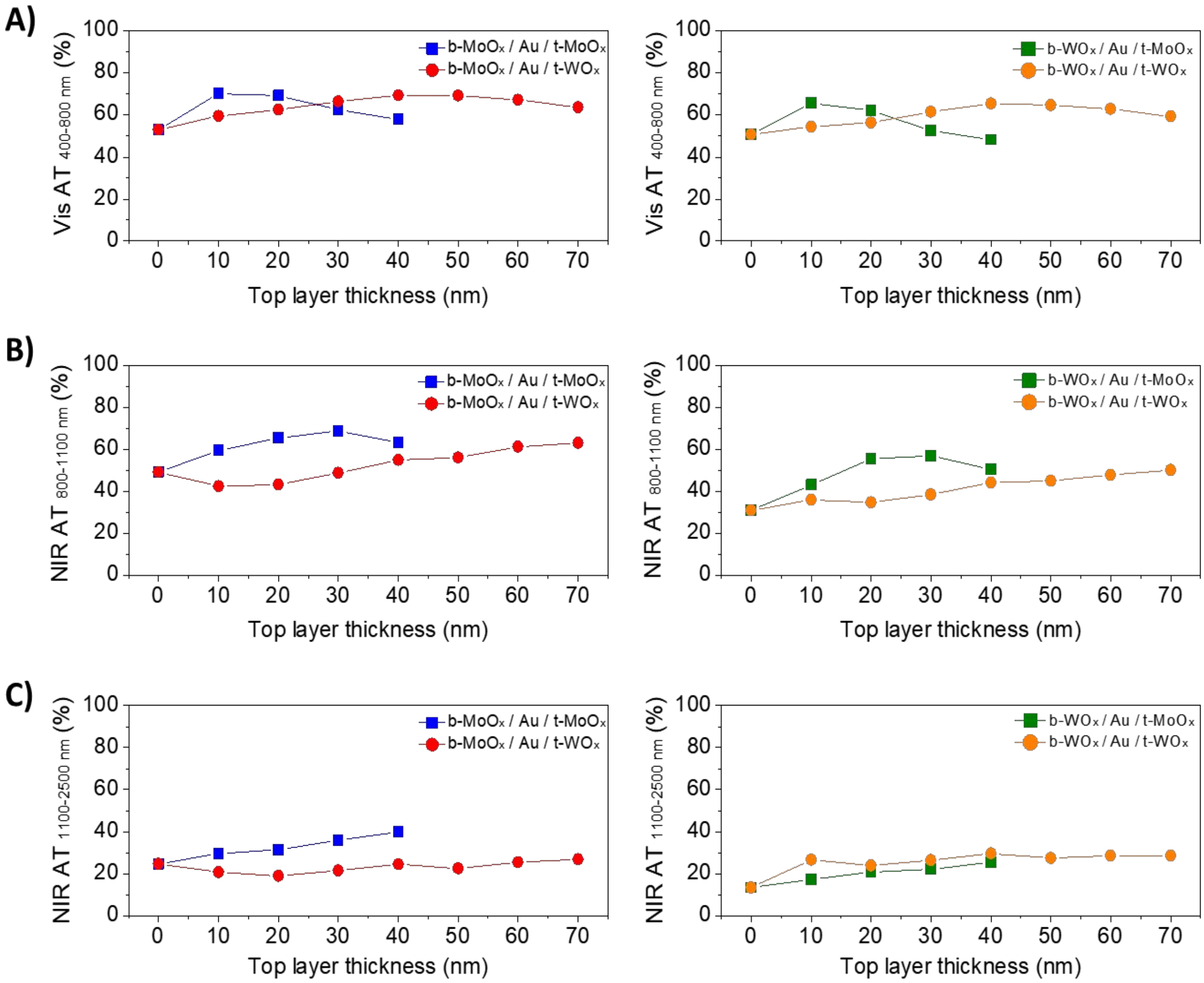

Figure S9. Average transmittance values calculated from the UV-Vis-NIR transmittance spectra in the spectral range A) 400-800nm ($AT_{400\text{-}800nm}$), B) 800-1100 nm ($AT_{800\text{-}1100nm}$) and C) 1100-2500 nm ($AT_{1100\text{-}2500nm}$) reported as a function of the top dielectric thickness for b-MoO_x/Au/t-MoO_x (blue), b-MoO_x/Au/t-WO_x (red), b-WO_x/Au/t-MoO_x (green), and b-WO_x/Au/t-WO_x (orange). All the b-D/Au/t-D multilayers have a 6 nm thick b-D and a 10 nm thick Au layer. The analysis of the different spectral ranges highlights that the 400-800 nm interval is mainly relevant for controlling visible transparency for applications in building integrated photovoltaic and smart windows, the 800–1100 nm range is important for tandem design with silicon-based solar cells, while the 1100–2500 nm region contributes to thermal management through the control of radiative heat exchange.

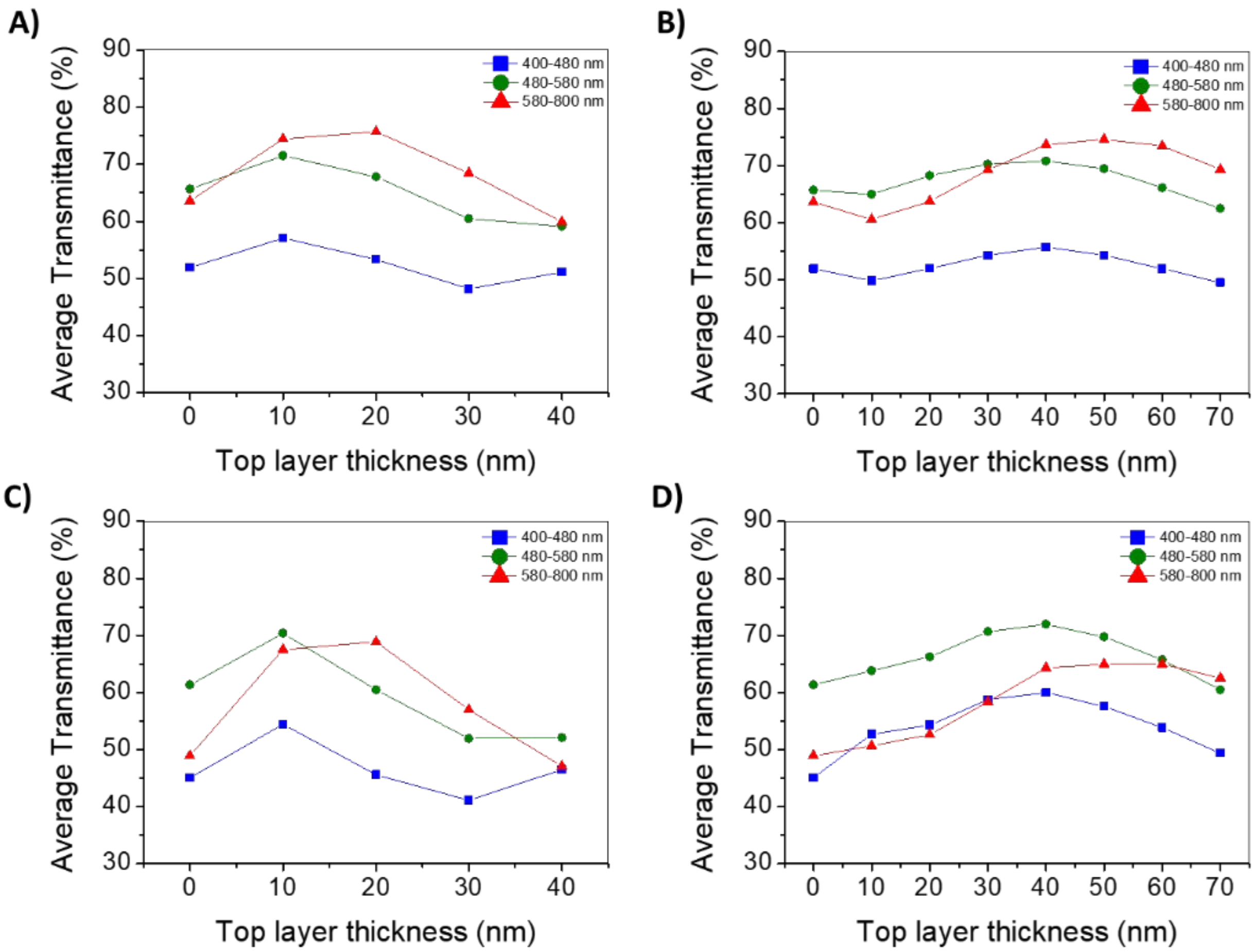

Figure S10. Average Transmittance of visible range divided in blue (400-480 nm), green (480-580 nm), and red (580-800 nm) of the b-D/Au/t-D as a function of t-D thickness for A) b-MoO_x/Au/t-MoO_x, B) b-MoO_x/Au/t-WO_x, C) b-WO_x/Au/t-MoO_x, and D) b-WO_x/Au/t-WO_x. The b-D thickness is 6 nm and Au is 10 nm for all the samples.

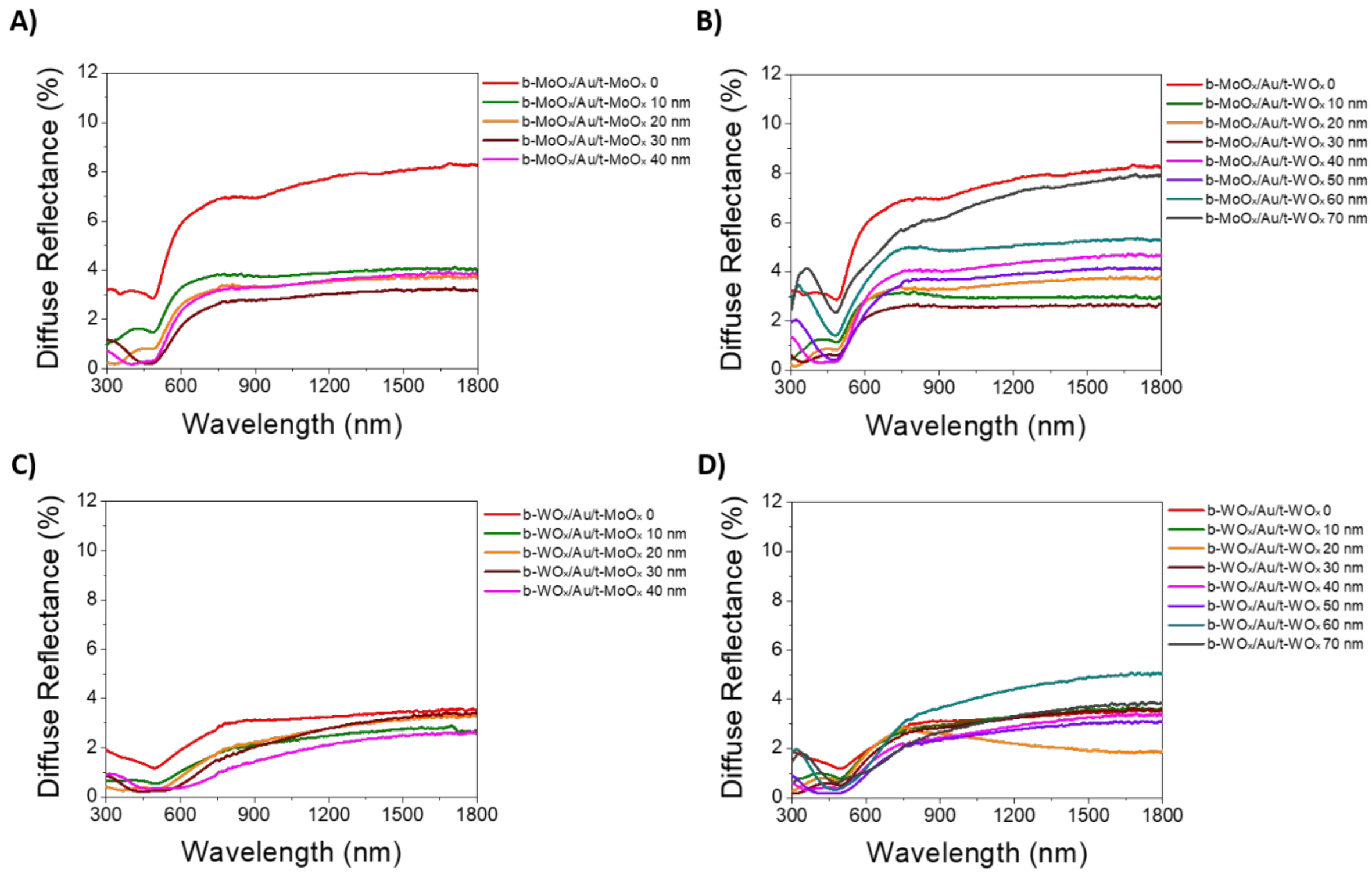

Figure S11. UV-Vis-NIR diffuse reflectance spectra within the spectral range 300–1800 nm of the b-D/Au/t-D as a function of t-D thickness for A) b-MoO_x/Au/t-MoO_x, B) b-MoO_x/Au/t-WO_x, C) b-WO_x/Au/t-MoO_x, and D) b-WO_x/Au/t-WO_x. For all the multilayers the thicknesses are 6 nm for b-D, 10 nm for Au, while the thickness of t-D is changed as reported in the labels.
